# Understanding the Effect of the Electron Spin Relaxation on the Relaxivities of Mn(II) Complexes with Triazacyclononane Derivatives

**DOI:** 10.1021/acs.inorgchem.1c02057

**Published:** 2021-10-07

**Authors:** Rocío Uzal-Varela, Laura Valencia, Daniela Lalli, Marcelino Maneiro, David Esteban-Gómez, Carlos Platas-Iglesias, Mauro Botta, Aurora Rodríguez-Rodríguez

**Affiliations:** † Centro de Investigacións Científicas Avanzadas (CICA) and Departamento de Química, Facultade de Ciencias, Universidade da Coruña, 15071, A Coruña, Galicia, Spain; ‡ Departamento de Química Inorgánica, Facultad de Ciencias, Universidade de Vigo, As Lagoas, Marcosende, 36310 Pontevedra, Spain; § Dipartimento di Scienze e Innovazione Tecnologica, Università del Piemonte Orientale “A. Avogadro”, Viale T. Michel 11, 15121 Alessandria, Italy; ∥ Departamento de Química Inorgánica, Universidade de Santiago de Compostela, Facultade de Ciencias, Campus de Lugo, 27002 Lugo, Galicia, Spain

## Abstract

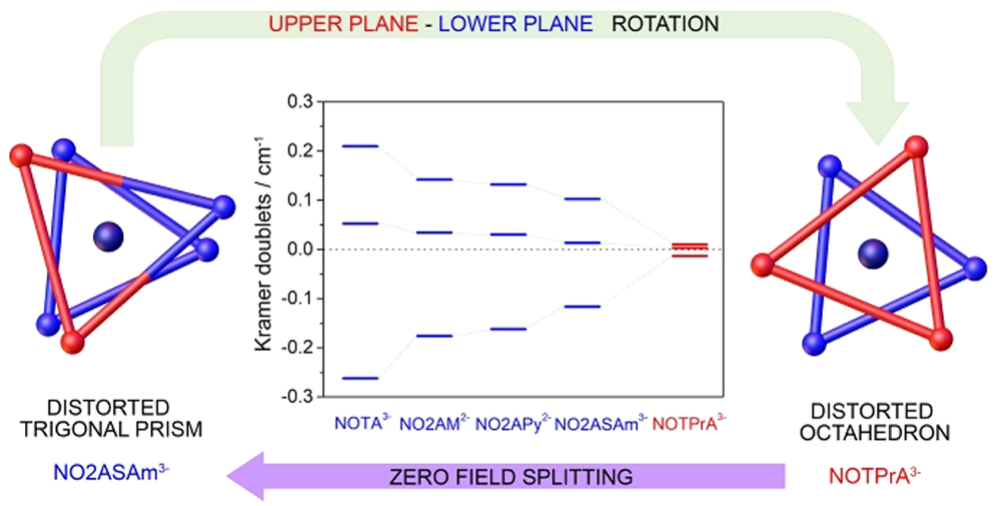

Investigating the relaxation of water ^1^H nuclei induced by paramagnetic Mn(II) complexes is important to understand the mechanisms that control the efficiency of contrast agents used in diagnostic magnetic resonance imaging (MRI). Herein, a series of potentially hexadentate triazacyclononane (TACN) derivatives containing different pendant arms were designed to explore the relaxation of the electron spin in the corresponding Mn(II) complexes by using a combination of ^1^H NMR relaxometry and theoretical calculations. These ligands include 1,4,7-triazacyclononane-1,4,7-triacetic acid (H_3_NOTA) and three derivatives in which an acetate group is replaced by sulfonamide (H_3_NO2ASAm), amide (H_2_NO2AM), or pyridyl (H_2_NO2APy) pendants. The analogue of H_3_NOTA containing three propionate pendant arms (H_3_NOTPrA) was also investigated. The X-ray structure of the derivative containing two acetate groups and a sulfonamide pendant arm [Mn(NO2ASAm)]^−^ evidenced six-coordination of the ligand to the metal ion, with the coordination polyhedron being close to a trigonal prism. The relaxivities of all complexes at 20 MHz and 25 °C (1.1–1.3 mM^–1^ s^–1^) are typical of systems that lack water molecules coordinated to the metal ion. The nuclear magnetic relaxation profiles evidence significant differences in the relaxivities of the complexes at low fields (<1 MHz), which are associated with different spin relaxation rates. The zero field splitting (ZFS) parameters calculated by using DFT and CASSCF methods show that electronic relaxation is relatively insensitive to the nature of the donor atoms. However, the twist angle of the two tripodal faces that delineate the coordination polyhedron, defined by the N atoms of the TACN unit (lower face) and the donor atoms of the pendant arms (upper face), has an important effect in the ZFS parameters. A twist angle close to the ideal value for an octahedral coordination (60°), such as that in [Mn(NOTPrA)]^−^, leads to a small ZFS energy, whereas this value increases as the coordination polyhedron approaches to a trigonal prism.

## Abstract 2

The nature of the donor atoms and particularly the coordination polyhedron affect dramatically the zero field splitting in six-coordinate Mn(II) complexes, which has a strong impact in the relaxivities measured at low magnetic fields.

## Introduction

Mn(II) complexes stable in aqueous media, in terms of dissociation and redox state, are currently the subject of intense research focused to find candidates as contrast agents for application in magnetic resonance imaging (MRI).^1−4^ MRI is an imaging technique used by radiologists to aid clinical diagnosis, as it provides high-resolution three-dimensional anatomical images.^5^ MRI detects the ^1^H NMR resonance of water molecules present in the body. The contrast in the acquired images is mainly related to differences in water proton density and in the relaxation times of water proton nuclei. Paramagnetic metal ions such as Gd(III), Mn(II), and Fe(III)^6−8^ are known to accelerate the relaxation rates of water ^1^H nuclei in their surroundings and thus can be used to enhance the image contrast.^9^ Most MRI scans are performed without administrating any contrast agent (around 60%).^10^ However, in many cases contrast-enhanced MRI is required to obtain more accurate information for the diagnosis of various diseases.^10^ This is nowadays achieved with the Gd(III) chelates available in the market, which are small complexes of this metal ion with polyamino–polycarboxylate ligands.^10^

A Mn(II)-based contrast agent was approved for clinical use already in 1997,^11,12^ but its utilization was discontinued,^13^ though the withdrawal from the market was not related to any safety concerns.^14^ Recent years witnessed a resurgence of interest in Mn(II) agents,^15−25^ which are expected to have better toxicity profiles compared with Gd(III) analogues.^26^ Gd(III) and Mn(II) complexes enhance water ^1^H relaxation following the same mechanism.^27^ The ^1^H relaxation enhancement (relaxivity) that they induce in aqueous solution is the result of both inner- and outer-sphere mechanisms, which depend on a number of structural and dynamic parameters of the complexes. Among them are the rotational correlation time of the complex (τ_R_), the mean residence time of the water molecule/s in the first coordination sphere (τ_m_), and the electronic relaxation times (*T*_*i*e_, *i* = 1 or 2), which affect both the inner- and outer-sphere contributions.^28^ Electron relaxation has a dominating contribution to relaxivity at low fields (<∼2 MHz), which are traditionally not used for clinical imaging. However, imaging at low fields is currently being explored as an alternative to high-field scanners, for instance, by applying variable fields (fast-field cycling MRI) and Mn(II) to generate contrast.^29^ Furthermore, the use of low-field scanners may have several advantages such as reduced image distortion, specific absorption rate and cost, or improved imaging near air–tissue interfaces.^30^ Given the growing interest in low- and ultralow MRI over the past years,^31^ an optimization of the relaxivities of contrast agents at low fields becomes of great interest.

Electron spin relaxation, according to the classical McLachlan theory, is promoted by transient distortions of the metal coordination environment that modulate the ZFS energy.^32^ More recent works on Gd(III) and Mn(II) complexes suggested that electron relaxation could have contributions from both the transient and static ZFS.^33−35^ However, the rational control of *T*_*i*e_ through ligand design remains a difficult task. Highly symmetrical coordination environments and ligand rigidity were found to favor slower electron relaxation in Gd(III) complexes,^36,37^ and these principles can be likely applied to Mn(II) chelates. For instance, the highly symmetrical [Mn(H_2_O)_6_]^2+^ complex presents a slow relaxation of the electron spin that results in very high relaxivities at low fields.^38^

**Scheme 1:**
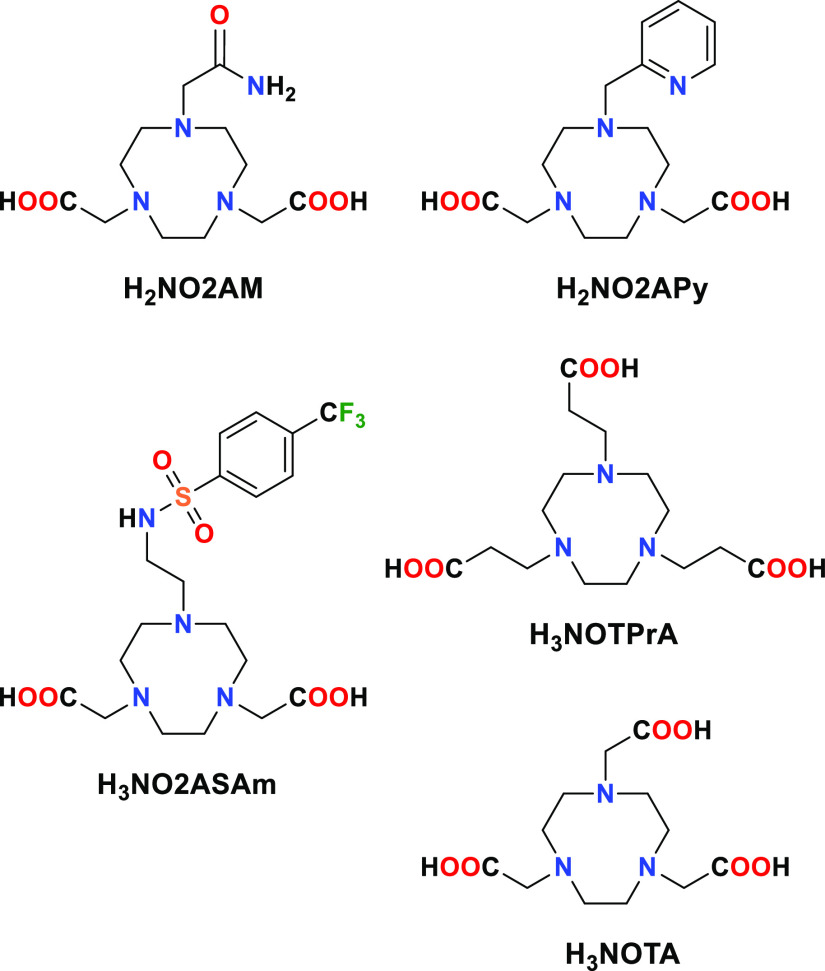
Chemical Structure of the Ligands Discussed in This Work

The work presented in this paper had a double aim. First, we wanted to investigate whether the classical paramagnetic relaxation theory provides reasonable ZFS energies for Mn(II) complexes. Second, we envisaged to analyze the factors affecting the ZFS in high-spin Mn(II) complexes, in particular the symmetry of the metal coordination environment. To this end, we decided to analyze the ^1^H nuclear relaxation dispersion (NMRD) profiles of a series of structurally related complexes derived from a tetraazacyclononane (TACN) macrocyclic platform. The complexes investigated include the well-known symmetric [Mn(NOTA)]^−^ complex^39^ and three derivatives in which one of the acetate pendant arms is replaced by a charged sulfonamide donor group (H_3_NO2ASAm)^40^ or neutral acetamide (H_2_NO2AM) or methylenepyridine (H_2_NO2APy) groups (Scheme 1). Furthermore, we also studied the complex with the propionic acid derivative H_3_NOTPrA,^41^ which maintains an identical donor set with respect to H_3_NOTA, but it is expected to modify the metal coordination environment. All these Mn(II) complexes are expected to lack water molecules in the first coordination sphere, and thus the observed ^1^H relaxivity involves outer-sphere contributions only. The ^1^H NMRD profiles were thus analyzed by using Freed’s outer-sphere model.^42^ The ZFS parameters obtained from this analysis are compared with those obtained with *ab initio* calculations based on CASSCF wave functions. The X-ray crystal structure of the [Mn(NO2ASAm)]^−^ complex is also reported.

## Results and Discussion

### Synthesis and X-ray Structure

The synthesis of the ligand H_3_NO2ASAm was reported in a previous work.^40^ Ligand H_2_NO2AM was prepared by alkylation of commercially available NO2A(O^*t*^Bu)_2_ with 2-chloroacetamide by using acetonitrile as a solvent and K_2_CO_3_ as a base. Subsequent hydrolysis of the *tert*-butyl esters under acidic conditions provided the ligand as the trifluoroacetate salt with 91% overall yield. The H_2_NO2APy and H_3_NOTPrA ligands were synthesized by alkylation of the NO2A(O^t^Bu)_2_ and TACN precursors with 2-(bromomethyl)pyridine and methyl 3-bromopropanoate in dry acetonitrile in the presence of K_2_CO_3_ as a base. Alkylation was followed by acid hydrolysis of the ester groups with HCl at room temperature. The ligands were isolated with an overall yield of 89% and 44%, respectively. The synthesis of the H_3_NOTPrA ligand was reported in the literature, following the alkylation of TACN with 3-bromopropanoic acid. However, the ligand could not be isolated in a pure form.^41^

**Figure 1:**
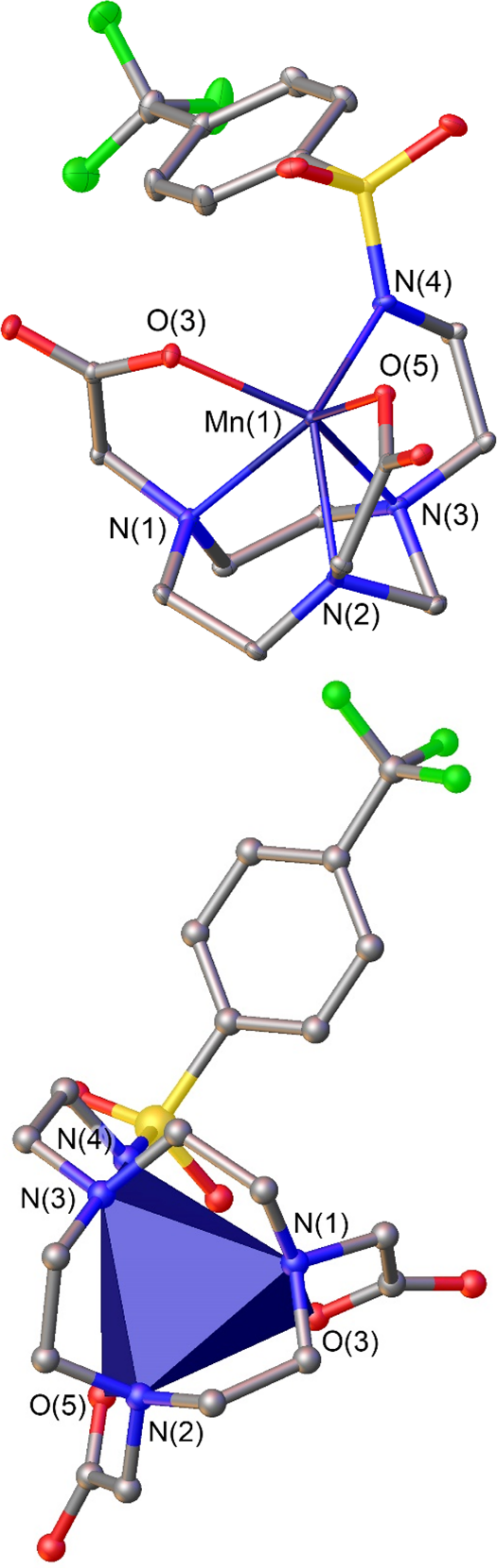
Top: structure of the [Mn(NO2ASAm)]^−^ complex present in crystals of {[Mn(NO2ASAm)]}_2_[Mn(H_2_O)_6_]·2H_2_O. Hydrogen atoms are omitted for simplicity. Bottom: view of the structure highlighting the trigonal prismatic coordination polyhedron. Bond distances of the metal coordination environment (Å): Mn(1)–O(3), 2.1303(8); Mn(1)–O(5), 2.1219(8); Mn(1)–N(1), 2.3560(10); Mn(1)–N(2), 2.3482(9); Mn(1)–N(3), 2.3331(10); Mn(1)–N(4), 2.1752(9).

The structure of the [Mn(NO2ASAm)]^−^ complex was determined with X-ray diffraction measurements (Figure 1). Crystals of formula {[Mn(NO2ASAm)]}_2_[Mn(H_2_O)_6_]·2H_2_O were obtained from an aqueous solution of the complex in the presence of excess Mn(II) chloride. Crystals contain the expected anionic [Mn(NO2ASAm)]^−^ complex, the octahedral [Mn(H_2_O)_6_]^2+^ complex, and water molecules. The [Mn(H_2_O)_6_]^2+^ complex shows a fairly regular octahedral coordination with Mn–O distances in the range 2.161–2.186 Å and an average distance of 2.174 Å. These distances are in good agreement with those observed previously in the solid state.^43^

The [Mn(NO2ASAm)]^−^ complex presents the expected structure in which the metal ion is coordinated by the three N donor atoms of the TACN unit, two oxygen atoms of carboxylate groups and the N atom of the sulfonamide pendant. The Mn–N distances involving the N atoms of the TACN fragment are similar to those observed for related six-coordinate Mn(II) complexes (2.23–2.41 Å).^44−49^ The distances to carboxylate oxygen atoms fall also within the expected range.^49,50^ The sulfonamide group coordinates through the nitrogen atom,^51,52^ and the Mn–N4 distance is ∼0.04 Å longer than those involving carboxylate oxygen atoms.

The coordination polyhedron in [Mn(NO2ASAm)]^−^ can be viewed as a twisted trigonal prism, where the two triangular faces are defined by N1, N2, and N3 (lower face, Figure 1) and O(3), O(5), and N(4) (upper face). These triangular faces are nearly parallel, intersecting at 1.6°. The Mn(II) ion is closer to the upper plane (0.977 Å) than to the plane defined by the three N atoms of the macrocycle (1.665 Å). The mean twist angle ϕ of the upper plane relative to the lower one amounts to 19.2°, which indicates that the coordination polyhedron is closer to a trigonal prism (ϕ = 0°) than to a trigonal antiprism (ϕ = 60°).

### Cyclic Voltammetry

The Mn(II) complexes were characterized by cyclic voltammetry measurements recorded from aqueous solutions of the complexes in 0.15 M NaCl. The [Mn(NOTA)]^−^ complex is characterized by an irreversible voltammogram with a half-wave potential *E*_1/2_ = 591 mV and Δ*E* = 274 mV (vs Ag/AgCl, scan rate 10 mV s^–1^). The separation of the anodic and cathodic waves increases dramatically upon increasing the scan rate (591 mV at 500 mV s^–1^, Figure S1 of the Supporting Information). This behavior suggests that the complex experiences an important rearrangement of the metal coordination sphere upon oxidation to Mn(III). The cyclic voltammograms of [Mn(NO2ASAm)]^−^, [Mn(NO2AM)], and [Mn(NOTPrA)]^−^ (scan rate 10 mV s^–1^) are typical of irreversible systems, showing oxidation peaks at 1011, 1138, and 1013 mV (Figure S2). The lack of reduction wave indicates that the Mn(II) species experiences a major structural change and/or chemical reaction upon oxidation (i.e., formation of hydroxide complexes). Indeed, irreversible Mn(II)/Mn(III) redox processes are rather common.^53^ This can be related to the lack of any ligand field stabilization energy (LFSE) in high-spin Mn(II) complexes, which results in coordination geometries determined by steric rather than electronic factors. Conversely, the LFSE in Mn(III) complexes results in a strong preference for octahedral coordination, with a consequent large inner-sphere contribution to electron transfer. Alternatively, the oxidation to Mn(III) may be accompanied by oxidative decarboxylation of acetate arms of the ligand, as observed for Ni(III) and Ce(IV) complexes.^54−56^ We notice that the oxidation potentials shift to more positive values following the sequence [Mn(NOTA)]^−^ < [Mn(NO2ASAm)]^−^ < [Mn(NO2AM)], as would be expected considering that Mn(III) is expected to be stabilized by hard donor groups. The oxidation potential is even more positive for [Mn(NO2APy)] and could not be determined under our conditions due to solvent discharge.

### pH Dependence of Proton Relaxivity (*r*_1p_)

**Figure 2:**
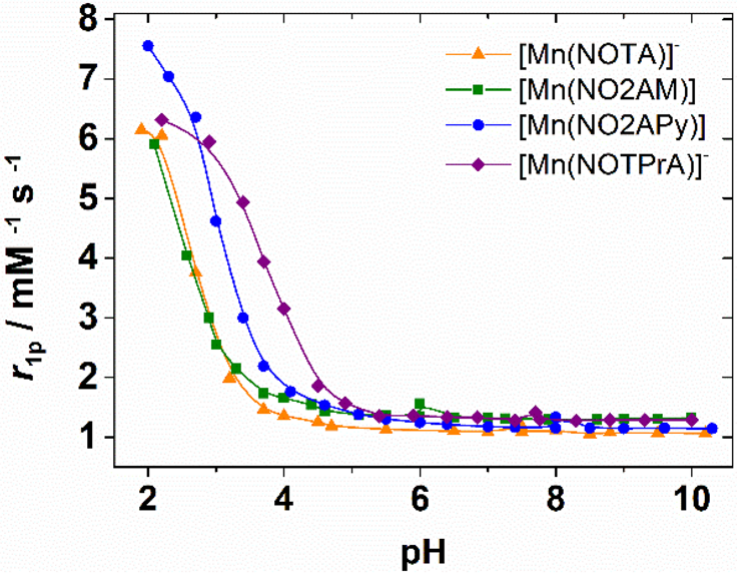
pH dependence of proton relaxivities (*r*_1p_) recorded at 298 K and 32 MHz.

The stability of the complexes in solution was assessed by measuring their relaxivities in aqueous solutions in the pH range ∼2.0–10.0 (Figure 2). The *r*_1p_ values recorded at 32 MHz and 298 K remain constant in a rather broad pH range up to pH ∼ 10.0. The relaxivities of [Mn(NOTA)]^−^, [Mn(NO2AM)], and [Mn(NO2APy)] increase below pH ∼ 4.5, reaching a relaxivity at pH 2.0 that is close to that of the [Mn(H_2_O)_6_]^2+^ complex (7.6 mM^–1^ s^–1^).^38^ Thus, this relaxivity increase at low pH can be attributed to complex dissociation. The dissociation of [Mn(NOTPrA)]^−^ takes place at a somewhat higher pH (<5.5), reflecting a lower stability of the complex. This is in line with previous studies, which pointed to a decreased complex stability upon replacement of acetate by propionate arms.^57^

### ^1^H NMRD Profiles

**Figure 3:**
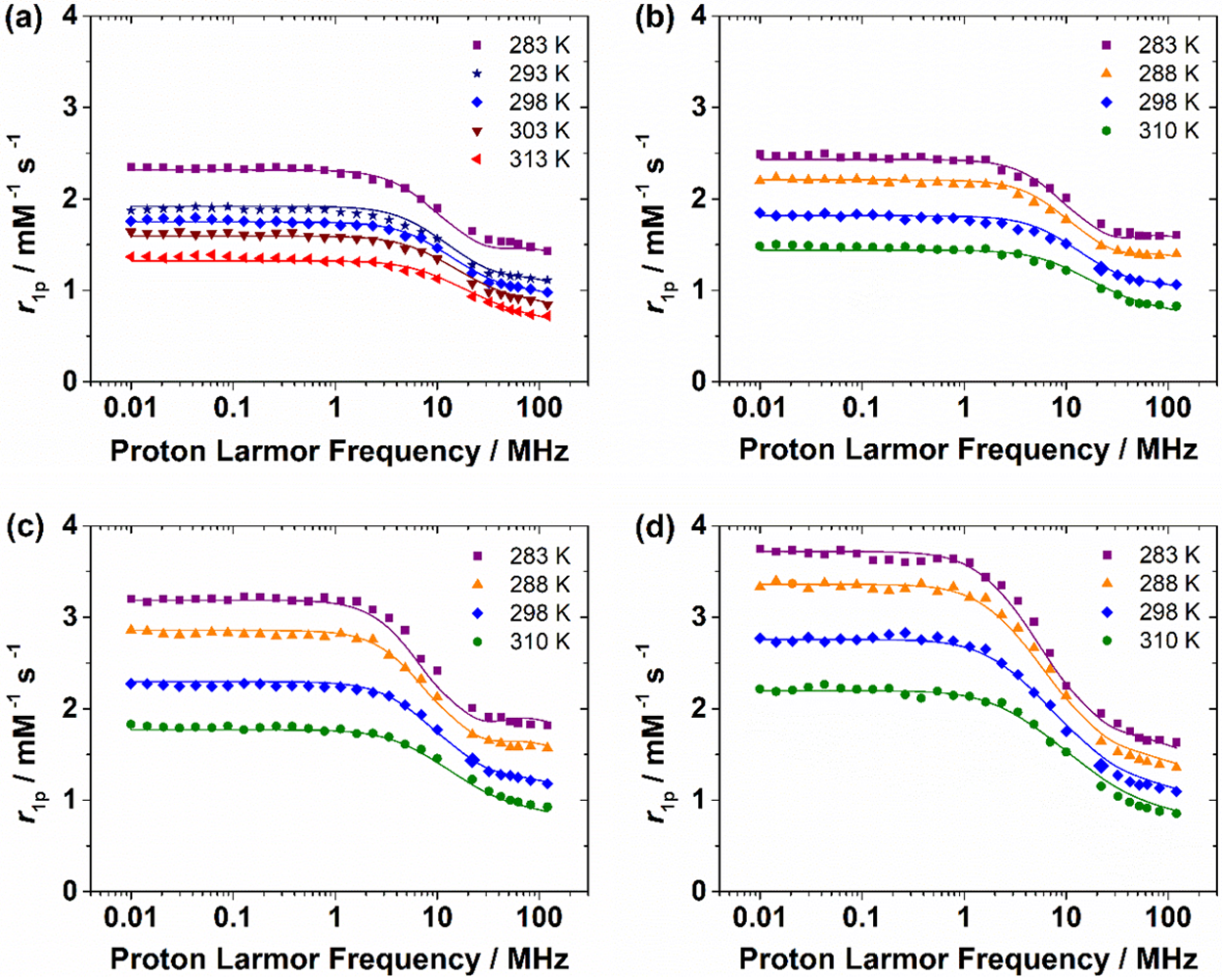
^1^H Nuclear magnetic relaxation dispersion (NMRD) profiles recorded at different temperatures for (a) [Mn(NOTA)]^−^, pH 7.4, 9.4 mM; (b) [Mn(NO2APy)], pH 7.0, 3.85 mM; (c) [Mn(NO2AM)], pH 7.0, 6.7 mM; and (d) [Mn(NOTPrA)]^−^, pH 7.5, 6.7 mM. The solid lines correspond to the fits of the data as described in the text.

The ^1^H NMRD profiles of the Mn(II) complexes were recorded at four or five different temperatures (283–313 K) in the ^1^H Larmor frequency range 9.97 × 10^–3^ to 120 MHz. The pH of the solutions used for NMRD studies was fixed in the range 7.0–8.0 to ensure full complex formation. The NMRD profiles (Figure 3) display one dispersion in the range 2–50 MHz, characteristic of Mn(II) complexes with low molecular weight.^58−60^ The lack of a second dispersion at lower fields (0.02–0.2 MHz) characteristic of the Mn(II) aqua complex demonstrates that the metal ion is fully complexed by all TACN derivatives.^50^ The relaxivities observed for the four complexes at 20 MHz and 25 °C (1.1–1.3 mM^–1^ s^–1^) are characteristic of complexes that do not contain coordinated water molecules.^61^ The hydration numbers estimated with the relaxivities observed at 0.01 MHz and 298 K take values of *q* < 0.45, confirming the absence of inner-sphere water molecules (Table S1).^62^

In the absence of coordinated water molecules, the observed relaxivity *r*_1p_ can be conveniently described by an outer-sphere model according to eq 1:^42^
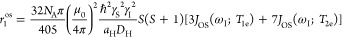
1Here *D*_H_ is the relative diffusion coefficient of the paramagnetic metal ion and water molecules, and *a*_H_ is the distance of closest approach of an outer-sphere water molecule to the paramagnetic center. The spectral density functions depend on the proton Larmor frequency ω_I_ and the relaxation times of the electron spin (*T*_1e_ and *T*_2e_) according to:
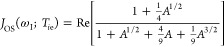
2
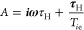
3
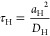
4

The diffusion coefficient *D*_H_ is generally assumed to obey an exponential law versus the inverse of temperature, with an activation energy *E*_D_:
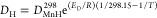
5The outer-sphere model defined by eqs 1–5, also called the Ayant–Belorizky–Hwang–Freed (ABHF) model,^63,64^ assumes that the water molecules and the Mn(II) complex are hard spheres diffusing in a viscous continuum, with the nuclear and electron spins located at the centers of these spheres. Thus, this model ignores the anisotropy of the molecules, the fact that the nuclear spin is not at the center of the water molecule, and potential attractive forces between water molecules and the complex (i.e., H-bonding interactions).^65^ Nevertheless, the ABHF model is commonly used to analyze the relaxivities of both Gd(III) and Mn(II) complexes.^66^ These limitations may introduce some errors in the electronic relaxation parameters obtained from the fits of the data, as electron spin has a minor yet non-negligible influence in the relaxivities at high magnetic fields (>20 MHz). However, we point out that the complexes investigated here do not contain coordinated water molecules and thus lack an inner-sphere contribution to relaxivity, which is also affected by electronic relaxation among several other dynamic parameters. Thus, the complexes investigated here represent an ideal family of structurally related Mn(II) derivatives for the accurate determination of their electronic relaxation parameters, within the limitations of the theoretical models commonly used to analyze relaxivity data.

**Figure 4:**
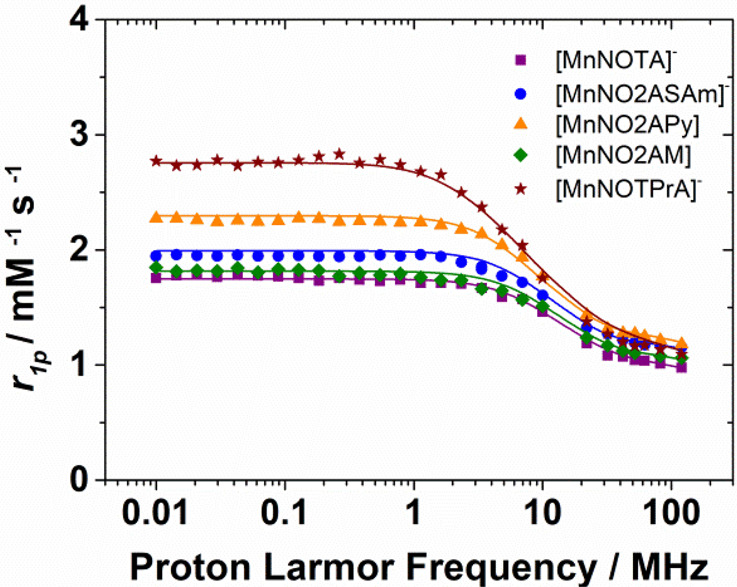
^1^H NMRD profiles recorded at 298 K.

The longitudinal and transverse electronic relaxation rates, 1/*T*_1e_ and 1/*T*_2e_, are often approximated by eqs 6 and 7, in which Δ^2^ is the mean-square ZFS energy and τ_V_ is the correlation time for the modulation of the zero-field-splitting interaction.^67^
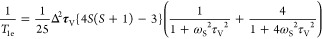
6
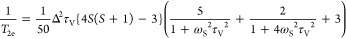
7The NMRD profiles recorded for the Mn(II) complexes investigated in this work evidence virtually identical relaxivities at high fields (>20 MHz, Figure 4). Under these conditions *T*_1e_ ≫ τ_H_, and thus the observed relaxivity is controlled by *D*_H_ and *a*_H_. These parameters are not expected to vary significantly for the series of structurally related complexes investigated here. However, the observed relaxivities show significant differences at low magnetic fields, where electron spin relaxation dominates. In particular, the *r*_1p_ values measured at low fields are very similar for [Mn(NOTA)]^−^, [Mn(NO2ASAm)]^−^, and [Mn(NO2APy)] but considerably higher for [Mn(NO2AM)] and particularly [Mn(NOTPrA)]^−^ (Table 1). These differences can only be explained by changes in the parameters that control the relaxation of the electron spin.

**Table 1:** Parameters Obtained from the Fits of ^1^H NMRD Data^a^

	Δ^2^ (10^20^ s^–2^)	*D*_MnH_^298^ (10^–10^ m^2^ s^–1^)	*E*_DMnH_ (kJ mol^–1^)	*r*_1p_ (0.01 MHz) (s^–1^ mM^–1^)
[Mn(NOTA)]^−^	2.4 ± 0.2	25.7 ± 0.1	20.5 ± 0.2	1.76
[Mn(NO2AM)]	1.7 ± 0.2	20.4 ± 0.1	23.3 ± 0.2	2.27
[Mn(NO2APy)]	2.5 ± 0.3	23.6 ± 0.1	22.0 ± 0.2	1.85
[Mn(NO2ASAm)]^−^	2.3 ± 0.2	21.4 ± 0.1	23.5 ± 0.3	1.95
[Mn(NOTPrA)]^−^	0.63 ± 0.06	22.0 ± 0.1	17.7 ± 0.2	2.77

a Parameters fixed during the fitting procedure: τ_V_ = 12 ps; *a*_H_ = 3.6 Å.

The ^1^H NMRD profiles were fitted to eqs 1–7 by using a nonlinear least-squares routine. Initial attempts to fit the data evidenced that Δ^2^ and τ_V_ are strongly correlated. Indeed, τ_V_ values in the range 8–14 ps afforded reasonably good fits of the data for all systems with sensibly different values of Δ^2^. We thus performed the fit of the data by fixing τ_V_ to 12 ps. Additionally, the value of *a*_H_ was fixed to 3.6 Å on the grounds of our previous experience.^61,68^ The fits of the data afforded the values of the diffusion parameters and Δ^2^, which are listed in Table 1. The values of *D*_H_ and *E*_D_ are very close to those characterizing the self-diffusion of water (*D*_H_ = 23.0 m^2^ s^–1^; *E*_D_ = 17.6 kJ mol^–1^).^69^ This is expected, as relative diffusion is dominated by the fast diffusion of water,^70^ and confirms that the value of *a*_H_ used for the fit is reasonable. The values of Δ^2^ estimated from the fits show the opposite sequence with respect to the relaxivities at low field, as would be expected. The [Mn(NOTA)]^−^, [Mn(NO2ASAm)]^−^, and [Mn(NO2APy)] complexes are characterized by very similar values of Δ^2^, while [Mn(NOTPrA)]^−^ presents the lowest value within this series of structurally related complexes. The Δ^2^ value obtained for [Mn(NOTPrA)]^−^ is virtually identical with that of the seven-coordinated [Mn(EDTA)]^2–^ complex, which contains a coordinated water molecule (Δ^2^ = 0.69 × 10^20^ s^–2^).^61^ Seven-coordinated Mn(II) complexes with pentagonal bipyramidal coordination geometries are, however, characterized by lower Δ^2^ values (Δ^2^ = 0.15 × 10^20^–0.6 × 10^20^ s^–2^),^71,72^ which shows that electron spin relaxation is affected by the coordination polyhedron.

### Optimized Geometries

The structures of the Mn(II) complexes were investigated by using DFT calculations (see the Computational Details section). The calculated bond distances involving the oxygen atoms of carboxylate groups (O_c_) and the sulfonamide nitrogen atom in [Mn(NO2ASAm)]^−^ are in excellent agreement with the X-ray values, with deviations <0.012 Å (Table 2). The calculated average distances to the nitrogen atoms of the macrocycle (N_am_) are overestimated by 0.06 Å. The [Mn(NOTA)]^−^ and [Mn(NOTPrA)]^−^ complexes display nearly undistorted *C*_3_ symmetries with rather similar Mn–N_am_ and Mn–O_c_ distances. However, these complexes show very different values of the angle ϕ characterizing the twist of the plane defined by the amine nitrogen atoms and that delineated by the donor atoms of the pendant arms. This angle takes a value of 18.8° for [Mn(NOTA)]^−^, indicating that the coordination polyhedron is close to a trigonal prism (0°). In the case of [Mn(NOTPrA)]^−^ (ϕ = 47.5°) the coordination polyhedron is best described as a trigonal antiprism (ideal value 60°).

**Table 2:** Bond Distances (Å) and Twist Angles (ϕ, deg) of the Mn(II) Coordination Spheres Obtained with DFT Calculations (TPSSh/Def-TZVPP)^a^

	Mn–N_am_	Mn–O_c_	Mn–X	ϕ
[Mn(NOTA)]^−^	2.370(0.001)	2.112(0.001)		18.8(0.1)
[Mn(NO2AM)]	2.361(0.031)	2.093(0.003)	2.189^b^	18.6(0.7)
[Mn(NO2APy)]	2.354(0.013)	2.094(0.004)	2.245^c^	18.7(0.6)
[Mn(NO2ASAm)]^−^	2.405(0.018)	2.117(0.001)	2.187^d^	17.9(2.4)
[Mn(NOTPrA)]^−^	2.361(0.001)	2.094(0.001)		47.5(0.1)

a Average values with standard deviations within parentheses.

b Distance to the oxygen atom of the amide group.

c Distance to the pyridine nitrogen atom.

d Distance to the sulfonamide nitrogen atom.

The substitution of one of the acetate arms of [Mn(NOTA)]^−^ by acetamide, sulfonamide, or pyridine groups introduces a certain distortion of the metal coordination geometry, as indicated by the calculated bond distances (Table 2). However, the values of ϕ remain nearly unaffected and close to that of [Mn(NOTA)]^−^.

The low value of Δ^2^ obtained from the fit of the NMRD profiles of [Mn(NOTPrA)]^−^ can be tentatively related to the trigonal antiprismatic metal coordination environment. However, it appears difficult to justify on the grounds of structural data the low Δ^2^ value of [Mn(NO2AM)] compared with [Mn(NOTA)]^−^, [Mn(NO2ASAm)]^−^, and [Mn(NO2APy)].

### Calculation of ZFS Parameters

**Figure 5:**
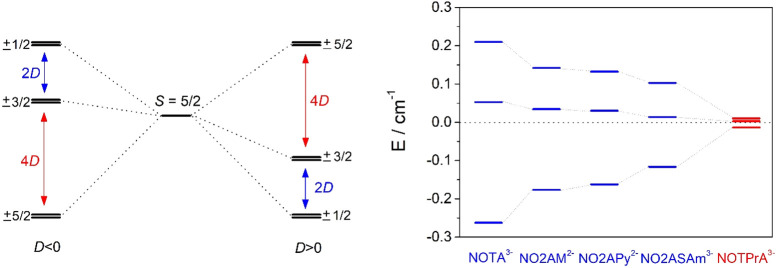
(left) ZFS of the Kramers doublets in symmetrical Mn(II) complexes for both *D* > 0 and *D* < 0. (right) Energies of the Kramers doublets calculated for the Mn(II) complexes investigated in this work at the CASSCF/NEVPT2 level.

For a paramagnetic high-spin Mn(II) complex with *S* = 5/2, the ZFS lifts the degeneration of the magnetic sublevels *M*_S_ = ±5/2, ±3/2, and ±1/2, generating three Kramers doublets (Figure 5). The axial (*D*) and rhombic (*E*) parts of the ZFS can be conveniently described within effective Hamiltonian theory as:
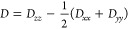
8
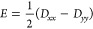
9Here, *D*_*xx*_, *D*_*yy*_, and *D*_*zz*_ are the principal components of the diagonalized *D* tensor. The ZFS energy Δ can be calculated from *D* and *E* via the relationship:
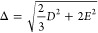
10

DFT methods were shown to have some limitations in the prediction of ZFS of metal complexes, the results being strongly dependent on the functional used and the amount of HF exchange.^73^ Previous studies demonstrated that the nonhybrid TPSS functional provided accurate ZFS parameters for Mn(II) and Gd(III) complexes.^34,74^ Wave function approaches based on the complete active space self-consistent field (CASSCF) method are nevertheless regarded to give more accurate ZFS parameters for transition metal complexes.^75^ Thus, we estimated the ZFS of the Mn(II) complexes investigated in this work by using both DFT and CAS(5,5) calculations (Table 3). This active space does not obviously take into account effects caused by ligand electrons. However, this appears to be a reasonable approximation considering the ionic nature of the metal–ligand bonds in the complexes investigated in this work and the absence of heavy atoms coordinated to the Mn(II) center (i.e., halide ligands).^76^ The values of *D* and *E* obtained from theoretical calculations were subsequently used to calculate Δ and Δ^2^ with eq 10.

**Table 3:** ZFS Parameters of Mn(II) Complexes Calculated Using DFT and *Ab Initio* Methods and Experimental Values Obtained from the Analysis of NMRD Profiles

					Δ^2^ (10^19^ s^–2^)	
		*D* (cm^–1^)	*E*/D	Δ (cm^–1^)	calcd	exptl
[Mn(NOTA)]^−^	TPSS	–0.0700	0.0014	0.0571	11.58	24
	CASSCF	–0.0410	0.0048	0.0335	3.98	
	NEVPT2	–0.0787	0.0108	0.0643	14.66	
[Mn(NO2AM)]	TPSS	–0.0760	0.0639	0.0624	13.83	17
	CASSCF	–0.0451	0.1729	0.0384	5.24	
	NEVPT2	–0.0529	0.0420	0.0433	6.65	
[Mn(NO2APy)]	TPSS	–0.0839	0.0076	0.0685	16.63	25
	CASSCF	–0.0485	0.0747	0.0399	5.66	
	NEVPT2	–0.0414	0.1387	0.0348	4.30	
[Mn(NO2ASAm)]^−^	TPSS	–0.0694	0.1016	0.0575	11.73	23
	CASSCF	–0.0343	0.1896	0.0295	3.08	
	NEVPT2	–0.0767	0.0943	0.0636	14.35	
[Mn(NOTPrA)]^−^	TPSS	–0.0144	0.0246	0.0118	0.492	6.3
	CASSCF	–0.0037	0.0326	0.0030	0.032	
	NEVPT2	–0.0039	0.0372	0.0032	0.036	

The calculated *D* values were found to be all negative at the DFT level, in agreement with the CASSCF results. The ZFS is the result of both the spin–orbit coupling (SOC) of excited states into the ground state and the direct electron–electron magnetic dipole spin–spin (SS) interaction involving unpaired electrons.^77^ The SS and SOC contributions to *D* calculated by using DFT (Table 4) evidence that the ZFS is largely dominated by SOC, with the SS part being responsible for ∼5.0–9.5% of the overall value of *D*. This is in sharp contrast with previous computational work, which pointed to a dominant role of the SS contribution in six-coordinate Mn(II) complexes containing neutral N/O donor atoms.^78^ The latter complexes were also characterized by positive *D* values, which again differs from the situation of the Mn(II) complexes presented in this work. However, coordination of an increasing number of negatively charged donor ligands was found to turn the sign of *D* negative.^79^

**Table 4:** Spin–Orbit Coupling (SOC) and Spin–Spin (SS) Contributions to the *D* Values (cm^–1^) Calculated with DFT

ligand	*D*_SOC_	*D*_SS_	α → α	β → β	α → β	β → α
NOTA	–0.0651	–0.0048	–0.0455	–0.0397	0.0036	0.0164
NO2AM	–0.0721	–0.0039	–0.0467	–0.0400	–0.0028	0.0175
NO2APy	–0.0789	–0.0049	–0.0257	–0.0155	–0.0404	0.0026
NO2ASAm	–0.0628	–0.0066	–0.0355	–0.0315	–0.0074	0.0116
NOTPrA	–0.0132	–0.0012	–0.0591	–0.0547	0.0684	0.0321

The α → α and β → β excitations, which maintain the spin multiplicity of the system, provide negative contributions to the *D* values. On the other hand, β → α excitations increase the electronic spin in one unit and generally provide a small positive contribution to *D*. Finally, α → β excitations present variable contributions within this series of structurally related complexes and are mainly responsible for the trend observed in the calculated *D* values. The [Mn(NO2APy)] complex shows a particularly large negative contribution of α → β excitations, presumably due to the weak coordination of the pyridyl group evidenced by the long calculated Mn–N_Py_ distance. Overall, the results shown in Table 4 evidence that the values of *D* are a subtle balance between contributions of d–d, ligand-to-metal, and metal-to-ligand charge transfer excitations, which appear to be quite sensitive to changes in just one of the donor atoms of the ligand.

The *E*/*D* values calculated by using DFT, CASSCF, and CASSCF/NEVPT2 calculations are lower than ∼0.19 cm^–1^. It has been shown that the prediction of the sign of *D* using computational methods becomes problematic for *E*/*D* > 0.22, at least by using DFT methods.^80^ Both positive and negative signs of *D* were obtained experimentally for six-coordinate Mn(II) complexes, with absolute values in the range 9 × 10^–4^ < *D* < 0.18 cm^–1^.^81^ However, the [Mn(MeNO2A)(H_2_O)] complex, which is closely related to those investigated here, displays a positive *D* value according to CASSCF/NEVPT2 calculations (+0.045 cm^–1^).^34^ These results highlight the difficulties of establishing relationships between the structure of Mn(II) complexes and the sign of *D*.

The inclusion of dynamic correlation effects using CASSCF/NEVPT2 calculations did not alter the sign of *D*, but their absolute values generally increased. This effect was observed previously, and it was related to the overestimation of the sextet–quartet excitation energies by CASSCF calculations.^80^ Indeed, the lowest sextet–quartet transition calculated for [Mn(NOTA)]^−^ at the CASSCF level [^4^E + ^4^A_2_(^4^G)] is 27902 cm^–1^, while this value reduces to 22462 cm^–1^ at the NEVPT2 level. The latter value is in excellent agreement with that estimated from the absorption spectrum (22173 cm^–1^, Figure S3). A similar situation is evidenced for [Mn(NOTPrA)]^−^, whose absorption spectrum evidence a feature at 20830 cm^–1^ attributable to the [^4^E + ^4^A_2_(^4^G)] ← ^6^A_1_ transitions. CASSCF calculations predict this absorption at 26638 cm^–1^, while NEVPT2 lowers this energy to 20912 cm^–1^, the latter being in satisfactory agreement with the experiment. The absorption spectrum of [Mn(NOTA)]^−^ presents a rather sharp feature at 23585 cm^–1^ that is typical of the [^4^E_g_ + ^4^A_1g_(^4^G)] ← ^6^A_1g_ excitations in octahedral complexes and was found to be rather insensitive to variations of the ligand field^82^ (24960 cm^–1^ for [Mn(H_2_O)_6_]^2+^).^83^ Our NEVPT2 calculations provide a calculated energy of 26504 cm^–1^, evidencing a significant overestimation with respect to the experimental value. However, NEVPT2 still considerably improves the agreement with the experiment compared with CASSCF (30813 cm^–1^).

The negative values of *D* can be explained by the energies of the Kramers doublets arising from a *S* = 5/2 spin system if *D* and *E* are defined such as 0 < *E*/*D* < 1/3 (Figure 5). The sign of *D* is expected to be negative when two of three Kramers doublets are higher in energy than the center of gravity,^80^ a situation that holds for all Mn(II) complexes investigated here at both the CASSCF and CASSCF/NEVPT2 levels.

The calculated ZFS parameters present similar values for the [Mn(NOTA)]^−^, [Mn(NO2AM)], [Mn(NO2APy)], and [Mn(NO2ASAm)]^−^ complexes. The values of Δ^2^ obtained with the calculated ZFS parameters are reasonably close to those obtained from the analysis of NMRD profiles. We notice that theoretical calculations fail to predict the slightly lower value of Δ^2^ determined for [Mn(NO2AM)]. Overall, the change of one of the donor atoms of the ligand does not have a dramatic effect in neither the experimental nor the calculated Δ^2^ values.

The ZFS parameters calculated for [Mn(NOTPrA)]^−^ differ dramatically from those of the remaining complexes of this series, which reflects a very different electronic structure. We note that the Δ^2^ value calculated with DFT is 1 order of magnitude higher than those obtained with CASSCF and CASSCF/NEVPT2 calculations. This discrepancy is not observed for the other Mn(II) complexes and appears to be related to the difficulties in predicting small values of *D*. Indeed, inspection of the data shown in Table 4 evidences that the individual contributions to *D* of the different types of excitations have absolute values higher than the overall value of *D*_SOC_. The small value of *D* is mainly the result of a partial cancellation of the negative contributions by α → α and β → β excitations and the positive values contributed by β → α and α → β excitations. Thus, small changes in the values of the individual contributions may significantly affect the calculated *D* value.

**Figure 6:**
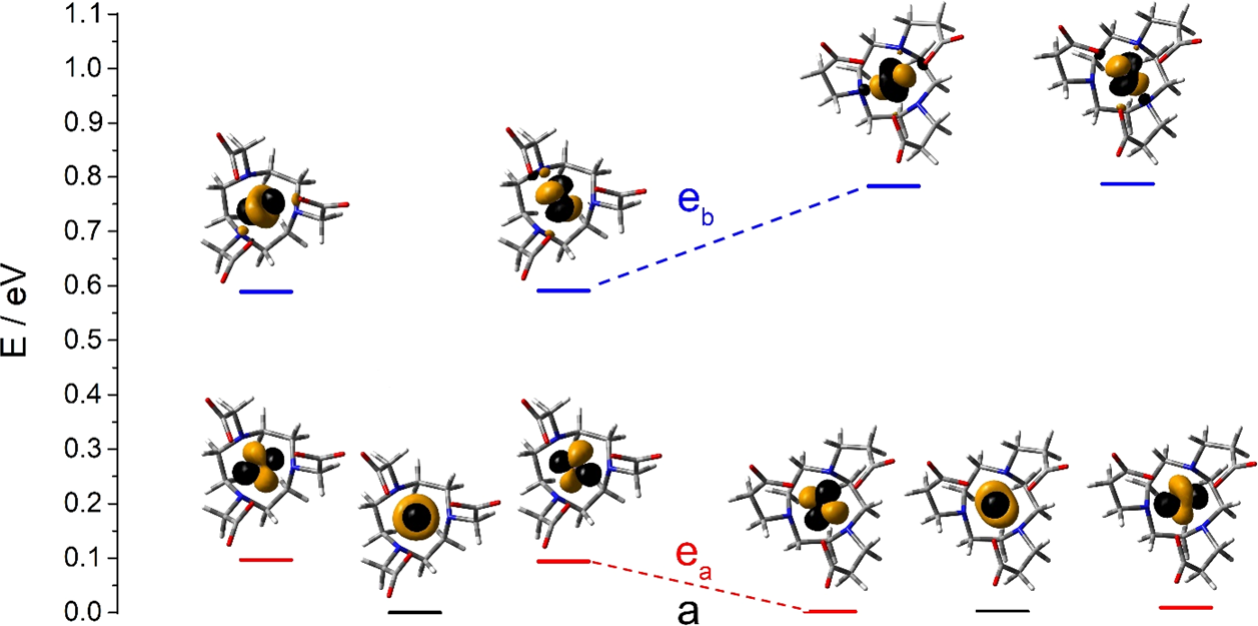
Splitting of the metal-based 3d orbitals obtained with AILFT calculations for [Mn(NOTA)]^−^ (left) and [Mn(NOTPrA)]^−^ (right). Symmetry labels are provided for the *C*_3_ point group. The molecules are oriented such that the *z*-axis matches the *C*_3_ symmetry axis.

The different ZFS parameters computed for [Mn(NOTPrA)]^−^ compared with the remaining Mn(II) complexes reported here can be traced back to the very different coordination environments discussed above. Indeed, the [Mn(NOTPrA)]^−^ complex is characterized by a pseudo-octahedral coordination environment evidenced by a twist angle of the N3 and O3 planes close to 60°. This is in nice agreement with the splitting of the metal 3d orbitals obtained with *ab initio* ligand field theory (AILFT) analysis based on CASSCF/NEVPT2 calculations (Figure 6). The AILFT analysis presented here transforms and orders the 3d-based CAS(5,5) active orbitals into the pure d-orbitals, providing the Racah parameters that account for interelectronic repulsion, the spin–orbit coupling constant ξ, and the ligand-field interaction expressed by a 5 × 5 one electron ligand field matrix.^84^ In *C*_3_ symmetry the five 3d orbitals classify into the *a* and *e* representations. The *a* and *e*_a_ sets correspond to the *t*_2g_ orbitals in octahedral symmetry, while *e*_b_ orbitals correspond to the *e*_g_ set.^85^ The LF orbitals calculated for [Mn(NOTPrA)]^−^ show that the *a* and *e*_a_ sets display very similar energies, while the *e*_b_ orbitals are roughly 0.8 eV higher in energy. The *a* orbital is essentially a d_*z*^2^_ orbital considering that the *z*-axis matches the *C*_3_ symmetry axis of the complex. The *e*_a_ orbitals present a major contribution of d_*xy*_ and d_*x*^2^–*y*^2^_ orbitals (63%) with a significant contribution of d_*xz*_ and d_*yz*_ (34%). The *e*_b_ orbitals present a major contribution of metal d_*xz*_ and d_*yz*_ orbitals (64%) and a lower contribution of d_*xy*_ and d_*x*^2^–*y*^2^_ (27%). In [Mn(NOTA)]^−^ the degeneration of the *a* and *e*_a_ orbitals is removed, yielding a splitting of the 3d orbitals characteristic of trigonal prismatic coordination.^86^ The *a* and *e*_a_ orbitals are separated by ∼0.1 eV, with the *e*_b_ orbitals being 0.59 eV higher in energy than the *a* orbital.

The data shown in Table 4 evidence that the origin of the ZFS parameters in these Mn(II) complexes is rather complex, which makes it difficult to make predictions based on simple rules. The same conclusion can be reached with CASSCF/NEVPT2 calculations by inspecting the individual contributions to the *D* tensor of the different quartet excited states (Table S2). The individual contributions of quartet states to *D* obtained for [Mn(NOTA)]^−^ vary in the range −0.56 to +0.69 cm^–1^, while for [Mn(NOTPrA)]^−^ they fall within the range −0.49 to +0.61 cm^–1^. The positive and negative contributions of the different excited quartet states compensate each other, resulting in absolute *D* values that are 1–2 orders of magnitude lower than the several individual contributions. An analogous situation was observed previously for Mn(II) complexes showing trigonal-bipyramidal coordination environments.^87^ In the case of [Mn(NOTPrA)]^−^ only six excited states provide the major contributions to *D*. Inspection of the corresponding wave functions shows that the d_*yz*_ ↔ d_*z*^2^_ excitation provides a positive contribution to *D*, while d_*yz*_ ↔ d_*xy*_, d_*xz*_ ↔ d_*z*^2^_, and d_*xz*_ ↔ d_*x*^2^–*y*^2^_ result in negative contributions (Table S2).

The energies calculated for the lowest-energy quartet state with NEVPT2 (before SOC) in the structurally related [Mn(NOTA)]^−^, [Mn(NO2ASAm)]^−^, [Mn(NO2AM)], and [Mn(NO2APy)] complexes are 22474, 22188, 21986, and 21610 cm^–1^. These values correlate with the oxidation potentials measured in aqueous solution of 728, 1011, 1138, and >1150 mV. The first excited quartet states calculated for these complexes display a major contribution from wave function (11), with weights in the range 0.53–0.65:
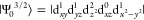
11Thus, the first quartet state is generated by a d_*xz*_ → d_*z*_^2^ spin flip excitation, and therefore one may expect a correlation with the oxidation potential, which involves removing an electron from a d_*xz*_ orbital.

The comparison of the Δ^2^ values computed theoretically and those obtained from the fits of the NMRD data must be taken with great caution. Indeed, calculations were performed on optimized structures and thus neglected any dynamic effects. Electron spin relaxation is known to present contributions from the transient and the static ZFS.^33,35^ The static ZFS corresponds to the average ZFS of all configurations present in solution. The transient ZFS is due to fluctuations of the metal coordination environment induced by vibrations and collisions with solvent molecules and corresponds to the spread of the ZFS energy. Thus, our calculations are expected to reflect better the static than the transient ZFS. The transient ZFS mechanism likely dominates the electronic relaxation in highly symmetrical systems like [Mn(H_2_O)_6_]^2+^, for which the static ZFS is very small. The [Mn(NOTPrA)]^−^ complex presents a small ZFS according to our theoretical calculations, which estimate a value of Δ^2^ that is 1–2 orders of magnitude lower than obtained with NMRD analysis. This suggests that the transient ZFS mechanism is the main responsible for electronic relaxation in this complex. Nevertheless, the NMRD data still evidence a slower electronic relaxation for [Mn(NOTPrA)]^−^ when compared to the other Mn(II) complexes investigated here. An estimate of the contributions of the transient and static ZFS contributions for a Gd(III) complex was performed by using *ab initio* molecular dynamics.^74,88^ Similar studies would be required to have a clearer picture of the mechanisms responsible for the electronic relaxation in the Mn(II) complexes investigated here.

Concerning the prediction of ZFS parameters using DFT and CASSCF/NEVPT2 calculations, the results reported in Table 3 show that the TPSS functional provides results that are comparable to, or even better than, those obtained with CASSCF/NEVPT2 calculations.

## Conclusions

The series of structurally related Mn(II) complexes investigated here allowed interrogating the factors that determine the relaxation of the electron spin. The combined use of ^1^H NMRD studies and theoretical calculations revealed that the rationalization of electron relaxation using simple rules remains difficult. However, the ZFS energies, and thus electron relaxation, appear to be more sensitive to the coordination polyhedron than to changes in the nature of the donor atoms. Theoretical calculations using both DFT and wave function approaches provide ZFS parameters in line with those derived from NMRD studies for trigonal prismatic complexes. This suggests that the classical description of electron relaxation of Solomon–Bloembergen–Morgan theory (eqs 6 and 7) is reasonably accurate for small Mn(II) complexes. Thus, we recommend the inclusion of the low field part of the NMRD profiles in the analysis of relaxometric data, in contrast to the common practice of some groups.^89^ For [Mn(NOTPrA)]^−^ theoretical calculations point to a very small ZFS, a situation that appears to be related to a coordination polyhedron closer to an octahedron. NMRD profiles evidence a considerably larger ZFS energy, most likely as a result of the transient ZFS mechanism. Overall, the results presented here represent a step forward toward the understanding of the relaxation mechanisms of potential Mn(II)-based MRI contrast agents. However, further studies are required to assess whether the conclusions of the present work can be generalized to complexes with different ligand families.

## Experimental Section

### Materials and Methods

Di-*tert*-butyl 2,2′-(1,4,7-triazonane-1,4-diyl) diacetate (NO2AO^*t*^Bu), 1,4,7-triazonane (TACN), and 2,2′,2″-(1,4,7-triazacyclononane-1,4,7-triyl)triacetic acid (NOTA) were purchased from CheMatech (Dijon, France). All other reagents were purchased from Aldrich Chemical Co. and used without further purification.

High-resolution electrospray ionization time-of-flight ESI-TOF mass spectra were recorded in the positive mode by using a LTQ-Orbitrap Discovery mass spectrometer coupled to a Thermo Accela HPLC. Medium performance liquid chromatography (MPLC) was performed by using a Puriflash XS 420 InterChim Chromatographer instrument equipped with a reverse phase Puriflash 15C18AQ column (60 Å, spherical 15 μm, 20 g) and UV-DAD detector, operating at a flow rate of 15 mL/min. Aqueous solutions were lyophilized by using a Biobase BK-FD10 Series apparatus. ^1^H and ^13^C NMR spectra of the ligands and their precursors were recorded at 298 K by using a Bruker AVANCE III 300, a Bruker AVANCE 400, or a Bruker AVANCE 500 spectrometer.

### Syntheses

#### Di-*tert*-butyl 2,2′-(7-(2-Amino-2-oxoethyl)-1,4,7-triazonane-1,4-diyl)diacetate (**1**)

A solution of 2-chloroacetamide (0.0158 g, 0.169 mmol) in dry CH_3_CN (4 mL) was added dropwise to a solution of di-*tert*-butyl 2,2′-(1,4,7-triazonane-1,4-diyl)diacetate (0.0603 g, 0.169 mmol) containing K_2_CO_3_ (0.0583 g, 0.422 mmol) in dry CH_3_CN (6 mL). The mixture was heated at 60 °C and stirred for 17 h. The reaction mixture was filtered, and the filtrate was evaporated to dryness in vacuo, giving a colorless oil (0.0694 g, 0.167 mmol, 99% yield). ^1^H NMR (400 MHz, CDCl_3_): δ 9.32 (b, 1H), 5.37 (b, 1H), 3.29 (s, 2H), 3.27 (s, 4H), 2.89–2.81 (m, 8H), 2.67 (t, *J* = 5.0 Hz, 4H), 1.45 (s, 18H). ^13^C NMR (101 MHz, CDCl_3_): δ 176.2, 171.3, 81.2, 61.0, 59.1, 56.6, 56.0, 55.3, 28.4. HRMS(ESI^+^): *m*/*z* calcd for C_20_H_39_N_4_O_5_ [M + H]^+^: 415.2915. Found: 415.2921.

#### H_2_NO2AM

Compound **1** (0.0680 g, 0.164 mmol) was dissolved in a mixture of CH_2_Cl_2_ and TFA (1:1) (10 mL), and the mixture was stirred at room temperature for 17 h. The acid was evaporated; water (4 × 4 mL) was added and evaporated again to remove most of the trifluoroacetic acid. The product was lyophilized to afford a yellow pale solid (0.0731 g, 0.154 mmol, 94% yield). ^1^H NMR (500 MHz, D_2_O, pH 1.64): δ 3.89 (s, 4H), 3.76 (s, 2H), 3.35 (s, 4H), 3.29 (t, *J* = 5.7 Hz, 4H), 3.18 (t, *J* = 5.7 Hz, 4H). ^13^C NMR (126 MHz, D_2_O, pH 1.46): δ 173.4, 171.9, 57.6, 56.5, 50.5, 50.0, 49.9. HRMS(ESI^+^): *m*/*z* calcd for C_12_H_23_N_4_O_5_ [M + H]^+^: 303.1663. Found: 303.1661. IR (ATR, υ̃ [cm^–1^]): 1728 and 1679 (C=O).

#### H_2_NO2APy

A solution of 2-(bromomethyl)pyridine (0.0440 g, 0.174 mmol) in dry CH_3_CN (10 mL) was added dropwise to a solution of di-*tert*-butyl 2,2′-(1,4,7-triazonane-1,4-diyl)diacetate (0.0622 g, 0.174 mmol) containing K_2_CO_3_ (0.0842 g, 0.609 mmol) in dry CH_3_CN (15 mL). The mixture was stirred at room temperature for 36 h. The reaction mixture was filtered, and the filtrate was evaporated to dryness in vacuo, giving a yellow oil that was used in the next step without further purification. The oil was dissolved in 3 M HCl (20 mL), and the mixture was stirred at room temperature for 20 h. The acid was evaporated, the residue was washed with water (3 mL), and the solvent was evaporated. The latter process was repeated twice to remove most of the acid. The product was lyophilized to afford a brown solid (0.0860 g, 0.155 mmol, 89% yield). ^1^H NMR (500 MHz, D_2_O, pH 1.234): δ 8.62 (d, *J* = 5.9 Hz, 1H), 8.50 (t, *J* = 7.9 Hz, 1H), 8.02 (d, *J* = 8.0, 1H), 7.94 (t, *J* = 7.9 Hz, 1H), 4.25 (s, 2H), 3.63 (s, 4H), 3.09 (d, *J* = 28.7 Hz, 8H), 2.77 (b, 4H). ^13^C NMR (126 MHz, D_2_O, pH 1.234): δ 173.4, 152.9, 147.2, 141.5, 128.0, 126.4, 56.3, 55.6, 50.2, 48.7, 47.4. HRMS(ESI^+^): *m*/*z* calcd for C_16_H_25_N_4_O_4_ [M + H]^+^: 337.1870. Found: 337.1871. IR (ATR, υ̃ [cm^–1^]): 1732 ν(C=O), 1617 ν(C=C).

#### Trimethyl 3,3′,3″-(1,4,7-triazonane-1,4,7-triyl)tripropionate (**2**)

A solution of methyl 3-bromopropanoate (0.4359 g, 2.610 mmol) in CH_3_CN (7 mL) was added dropwise to a solution of 1,4,7-triazonane (0.1022 g, 0.7910 mmol) containing K_2_CO_3_ (0.8199 g, 5.933 mmol) in CH_3_CN (10 mL). The mixture was heated at 60 °C and stirred for 89 h. The reaction mixture was filtered, and the filtrate was evaporated to dryness in vacuo, giving a yellow oil (0.2560 g, 0.6607 mmol, 84% yield). ^1^H NMR (300 MHz, CDCl_3_): δ 3.66 (s, 9H), 2.84 (t, *J* = 7.1 Hz, 6H), 2.71 (b, 12H), 2.54 (t, *J* = 7.1 Hz, 6H) ^13^C NMR (75.0 MHz, CDCl_3_): δ 173.2, 55.2, 54.0, 51.5, 33.2. HRMS(ESI^+^): *m*/*z* calcd for C_18_H_33_N_3_O_6_ [M + H]^+^: 388.2442. Found: 388.2444.

#### H_3_NOTPrA

Compound **2** (0.2418 g, 0.6240 mmol) was dissolved in 6 M HCl (30 mL), and the mixture was refluxed for 20 h. The acid was evaporated; water (3 × 3 mL) was added and evaporated again to remove most of the acid. The product was purified by MPLC on reverse phase (H_2_O:CH_3_CN; compound eluted at 62% CH_3_CN) and lyophilized to afford a yellow pale solid (0.1199 g, 0.3471 mmol, 44% yield). ^1^H NMR (500 MHz, D_2_O, pH 0.58): δ 3.28 (t, *J* = 6.9 Hz, 6H), 3.22 (s, 12H), 2.64 (t, *J* = 6.9 Hz, 6H). ^13^C NMR (126 MHz, D_2_O, pH 0.58): δ 175.2, 52.4, 49.2, 28.8. HRMS(ESI^+^): *m*/*z* calcd for C_15_H_28_N_3_O_6_ [M + H]^+^: 346.1973. Found: 346.1971. IR (ATR, υ̃ [cm^–1^]): 1706 (C=O).

#### Synthesis of the Complexes

All complexes were prepared *in situ* by mixing appropriate amounts of the ligand and MnCl_2_·4H_2_O and subsequent adjustment of the pH with diluted aqueous NaOH and HCl solutions.

### Computational Details

The geometries of the Mn(II) complexes were optimized with the Gaussian16 program package^90^ by using the hybrid meta-GGA TPSSh^91^ exchange-correlation functional and the Def2-TZVPP^92^ basis set. Bulk solvent effects were incorporated by using the polarized continuum model with the default settings implemented in G16.^93^ The size of the integration grid was increased with the integral = superfinegrid keyword. Frequency calculations were used to confirm that the optimized geometries corresponded to local energy minima on the potential energy surface.

The optimized geometries were used for state averaged complete active space self-consistent field (SA-CASSCF)^94−96^ calculations, which were performed by using the ORCA4 program (ver. 4.2.0).^97,98^ The super-CI-PT algorithm was used for the iterative orbital update procedure.^99^ The active space included the five 3d electrons of Mn(II) distributed over the five metal-based d orbitals [CAS(5,5)] by using 1 sextet, 24 quartet, and 75 doublet roots. These calculations employed the Def2-TZVPP^92^ basis set and were accelerated by introducing the resolution of identity (RIJK)^100^ approximation with the aid of the Def2/JK^101^ auxiliary basis set. Dynamic correlation was considered with the fully internally contracted variant of *N*-valence state perturbation theory (FIC-NEVPT2)^102−104^ using the RIJCOSX^105^ approximation and the Def2-JK^101^ auxiliary basis set. Spin–orbit coupling was introduced in the framework of quasi-degenerate perturbation theory (QDPT).^106^*Ab initio* ligand field (AILF) calculations were performed with the method proposed by Atanasov, as implemented in ORCA.^107^ ZFS parameters were also calculated at the TPSS/Def2-TZVPP level by using the coupled-perturbed method^108^ to estimate the SOC contribution and the spin-unrestricted natural orbital (UNO) determinant to obtain the spin–spin contribution.^109^ Spin–orbit effects were included by using the mean-field approach SOMF(1X).^110^ Solvent effects (water) in ORCA calculations were considered with Truhlar’s universal solvation model, which is based on solute electron density and on a continuum model of the solvent (SMD).^111^

### Relaxometric Measurements

1/*T*_1_^1^H nuclear magnetic relaxation dispersion (NMRD) profiles were acquired with two different instruments. Low field data from 9.97 × 10^–3^ to 10 MHz proton Larmor frequency were measured by using a fast-field cycling (FFC) Stelar SmarTracer relaxometer (Stelar s.r.l., Mede, PV, Italy) equipped with a silver magnet, with an uncertainty in 1/*T*_1_ of ca. 1%. The points corresponding to high field strengths (20–120 MHz proton Larmor frequency) were collected with a high field relaxometer (Stelar) equipped with the HTS-110 3T Metrology cryogen-free superconducting magnet. The measurements were performed by using the standard inversion recovery sequence (20 experiments, 2 scans) with a typical 90° pulse width of 3.5 μs, and the reproducibility of the data was within ±0.5%. The temperature was controlled with a Stelar VTC-91 heater airflow equipped with a copper–constantan thermocouple (uncertainty of ±0.1 K).

The Mn(II) complexes were prepared by mixing solutions of MnCl_2_ and the corresponding ligand by using an ∼5% molar excess of the ligand to avoid the presence of free Mn(II) in solution. The pH was adjusted to ∼7.0 with HCl or NaOH. The concentration of Mn(II) complexes was evaluated by ^1^H NMR measurements (Bruker Avance III spectrometer equipped with a wide bore 11.7 T magnet) using the well-established Evans’s method.^112^

The variation of the longitudinal relaxation rate as a function of pH was measured on the Mn(II) complexes in the range 2–10, at 32 MHz and 298 K. Every sample (with initial neutral pH) was divided into two aliquots, which were used to measure the pH dependence in the acidic (7–2) and basic ranges (7–10). The pH was adjusted by using negligible volumes of diluted HCl/NaOH to decrease/increase the pH of the acidic/basic ranges, while keeping the concentration of the complex constant during the experiments. After reaching the end of the titrations, the pH was brought back to neutrality to verify the reversibility of the process.

### Cyclic Voltammetry Measurements

Electrochemical measurements were performed by using an Autolab PGSTAT101 potentiostat using a three-electrode configuration. The working electrode was a glassy carbon (Metrohm 6.1204.000) disc while a Pt wire and an Ag/AgCl (Metrohm 6.0728.000) electrode served as counter and reference electrodes, respectively. The Ag/AgCl electrode was filled with 3 M KCl. Measurements were made with ∼2 × 10^–3^ M solutions of complexes, prepared *in situ* from manganese(II) chloride and the corresponding ligand, in distilled water at pH 6.3–7.0, with 0.15 M sodium chloride as a supporting electrolyte. The solutions were deoxygenated before each measurement by bubbling N_2_. The glassy carbon working electrode was mechanically cleaned before each experiment by polishing its surface using a polishing kit (Metrohm 6.2802.010), first with α-Al_2_O_3_ (0.3 μm) and after washed with purified water.

### X-ray Diffraction Measurements

A single crystal of {[Mn(NO2ASAm)]}_2_[Mn(H_2_O)_6_]·2H_2_O was analyzed by X-ray diffraction. Table 5 shows the crystallographic data and the structure refinement parameters. Crystallographic data were collected at 100 K by using a Bruker D8 Venture Photon II CMOS detector and Cu Kα radiation (λ = 1.54178 Å) generated by an Incoatec high brillance microfocus source equipped with Incoatec Helios multilayer optics. The software APEX3^113^ was used for collecting frames of data, indexing reflections, and the determination of lattice parameters, SAINT^114^ for integration of the intensity of reflections, and SADABS^115^ for scaling and empirical absorption correction. The SHELXT program^116^ was used for solving the structure by dual-space methods while the SHELXL-2018/3 program^117^ was used for refining all non-hydrogen atoms with anisotropic thermal parameters by full-matrix least-squares calculations on *F*^2^. Most hydrogen atoms of the compound were inserted at calculated positions and constrained with isotropic thermal parameters. However, the hydrogen atoms of the [Mn(H_2_O)_6_]^2+^ complex were located from a Fourier-difference map and refined isotropically.

**Table 5:** Crystal Data and Structure Refinement Details

parameter	value
formula	C_19_H_32_F_3_Mn_1.5_N_4_O_10_S
molecular weight, MW	647.95
crystal system	triclinic
space group	*P*-1
*a*	7.6677(3) Å
*b*	9.2366(4) Å
*c*	19.8388(9) Å
α	91.376(2)°
β	94.319(2)°
γ	108.882(1)°
*V*	1323.92(10) Å^3^
*F*(000)	669.0
Z	2
*D*_calc_	1.625 g cm^–3^
μ	0.885
θ range	2.33°–28.29°
*R*_int_	0.0251
measured reflections	63585^a^
goodness of fit, GOF on *F*^2^	1.053
*R*_1_	0.0238
*wR*_2_ (all data)	0.0599
largest differences	0.492 e Å^–3^ (peak), −0.477 e Å^–3^ (hole)

a Of which 6572 were independent and 6370 were unique, with *I* > 2σ(*I*).

## Supporting Information Available

NMR and MS of ligands and their precursors, UV–vis absorption spectra, additional relaxometric data, cyclic voltammograms, contributions to *D* obtained with NEVPT2 calculations, Cartesian coordinates obtained with DFT calculations (PDF)

## Accession Codes

CCDC 2089755 contains the supplementary crystallographic data for this paper. These data can be obtained free of charge via www.ccdc.cam.ac.uk/data_request/cif, or by emailing data_request@ccdc.cam.ac.uk, or by contacting The Cambridge Crystallographic Data Centre, 12 Union Road, Cambridge CB2 1EZ, UK; fax: + 44 1223 336033.
