# Supplementary material for: Understanding the Effect of the Electron Spin Relaxation on the Relaxivities of Mn(II) Complexes with Triazacyclononane Derivatives
Source: Inorg Chem. 2021 Oct 7;60(20):15055–68. doi: 10.1021/acs.inorgchem.1c02057 (PMC8527457; doi:10.1021/acs.inorgchem.1c02057)
Supplement: Supplementary file 1 — ic1c02057_si_001.pdf [file ic1c02057_si_001.pdf]

Supporting Information for:

Understanding the effect of the electron spin relaxation on the relaxivities of Mn(II) complexes with triazacyclononane derivatives

Rocío Uzal-Varela,[a] Laura Valencia,[b] Daniela Lalli,[c] Marcelino Maneiro,[d] David Esteban-Gómez,[a] Carlos Platas-Iglesias,[a] Mauro Botta*[c] and Aurora Rodríguez-Rodríguez*[a]

[a] Universidade da Coruña, Centro de Investigacións Científicas Avanzadas (CICA) and Departamento de Química Fundamental, Facultade de Ciencias, 15071, A Coruña, Galicia, Spain.

[b] Departamento de Química Inorgánica, Facultad de Ciencias, Universidade de Vigo, As Lagoas, Marcosende, 36310 Pontevedra, Spain.

[c] Dipartimento di Scienze e Innovazione Tecnologica, Università del Piemonte Orientale "A. Avogadro", Viale T. Michel 11, 15121 Alessandria, Italy.

[d] Departamento de Química Inorgánica, Universidade de Santiago de Compostela, Facultade de Ciencias, Campus de Lugo, 27002 Lugo, Galicia, Spain.

Email: aurora.rodriguez@udc.es (A. R.-R.)
Email: mauro.botta@uniupo.it (M. B.)

Summary

Figure S1. Cyclic voltammogram of **[Mn(NOTA)]$^-$** complex in aqueous solution in 0.15 M NaCl, recorded at 10, 50, 100, 250, 400 and 500 mV s^{-1}. 4

Figure S2. Cyclic voltammogram of **[Mn(NO2ASAm)]$^-$**, **[Mn(NOTPrA)]$^-$** and **[Mn(NO2AM)]** complexes in aqueous solution in 0.15 M NaCl, recorded at 10 mV s^{-1}. 4

Figure S3. Solid line: Absorption spectrum of a 0.1015 M solution of the Mn^{2+} complex of **NOTA^{3-}** recorded at 298 K. Dashed line: Absorption spectrum of a 0.1006 M solution of the Mn^{2+} complex of **NOTPrA^{3-}** recorded at 298 K. 5

Figure S4. ^{1}H NMR spectrum of compound **1** (400 MHz, $CDCl_3$, 298 K). 6

Figure S5. ^{13}C NMR spectrum of compound **1** (101 MHz, $CDCl_3$, 298 K). 6

Figure S6. Experimental high resolution mass spectrum (ESI$^+$) of compound **1**. 7

Figure S7. ^{1}H NMR spectrum of **H$_2$NO2AM** (500 MHz, D_2O, pH 1.64, 298 K). 8

Figure S8. ^{13}C NMR spectrum of **H$_2$NO2AM** (126 MHz, D_2O, pH 1.64, 298 K). 8

Figure S9. Experimental high resolution mass spectrum (ESI$^+$) of **H$_2$NO2AM**. 9

Figure S10. ^{1}H NMR spectrum of **H$_2$NO2APy** (500 MHz, D_2O, pH 1.23, 298 K). 10

Figure S11. ^{13}C NMR spectrum of **H$_2$NO2APy** (126 MHz, D_2O, pH 1.23, 298 K). 10

Figure S12. Experimental high resolution mass spectrum (ESI$^+$) of **H$_2$NO2APy**. 11

Figure S13. ^{1}H NMR spectrum of compound **2** (300 MHz, $CDCl_3$, 298 K). 12

Figure S14. ^{13}C NMR spectrum of compound **2** (75.5 MHz, $CDCl_3$, 298 K). 12

Figure S15. Experimental high resolution mass spectrum (ESI$^+$) of compound **2**. 13

Figure S16. ^{1}H NMR spectrum of **H$_3$NOTPrA** (500 MHz, D_2O, pH 0.58, 298 K). 14

Figure S17. ^{13}C NMR spectrum of **H$_3$NOTPrA** (126 MHz, D_2O, pH 0.58, 298 K). 14

Figure S18. Experimental high resolution mass spectrum (ESI$^+$) of **H$_3$NOTPrA**. 15

Table S1. Hydration numbers estimated with the relaxivities observed at 0.01 MHz and 298 K. 16

Table S2. Individual contributions to the *D* values obtained with NEVPT2 calculations for **[Mn(NOTA)]$^-$**. 16

Table S3. Major individual contributions to the *D* values obtained with NEVPT2 calculations for **[Mn(NOTPrA)]$^-$**. 17

Table S4. Optimized Cartesian coordinates obtained for **[Mn(NOTA)]$^-$** (TPSSh/Def2-TZVPP, scrf=pcm). 18

Table S5. Optimized Cartesian coordinates obtained for **[Mn(NOTPrA)]**$^-$ (TPSSh/Def2-TZVPP, scrf=pcm)... 19

Table S6. Optimized Cartesian coordinates obtained for **[Mn(NO2AM)]** (TPSSh/Def2-TZVPP, scrf=pcm)... 21

Table S7. Optimized Cartesian coordinates obtained for **[Mn(NO2APy)]** (TPSSh/Def2-TZVPP, scrf=pcm)... 22

Table S8. Optimized Cartesian coordinates obtained for **[Mn(NO2ASAm)]**$^-$ (TPSSh/Def2-TZVPP, scrf=pcm)... 24

Table S9. Sample ORCA input file.. 26

Table S10. Bond distances (Å) and angles (°) of the metal coordination environment in {**[Mn(NO2ASAm)]**}$_2$**[Mn(H$_2$O)$_6$]·2H$_2$O**... 28

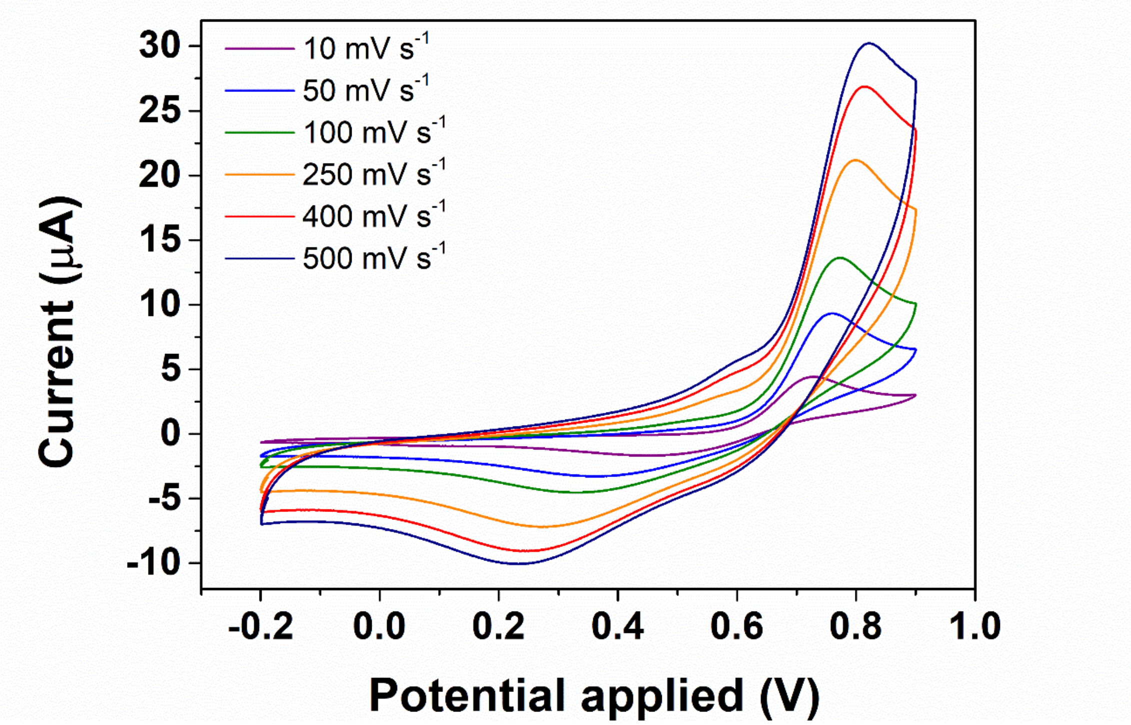

Figure S1. Cyclic voltammogram of **[Mn(NOTA)]⁻** complex in aqueous solution in 0.15 M NaCl, recorded at 10, 50, 100, 250, 400 and 500 mV s^{-1}.

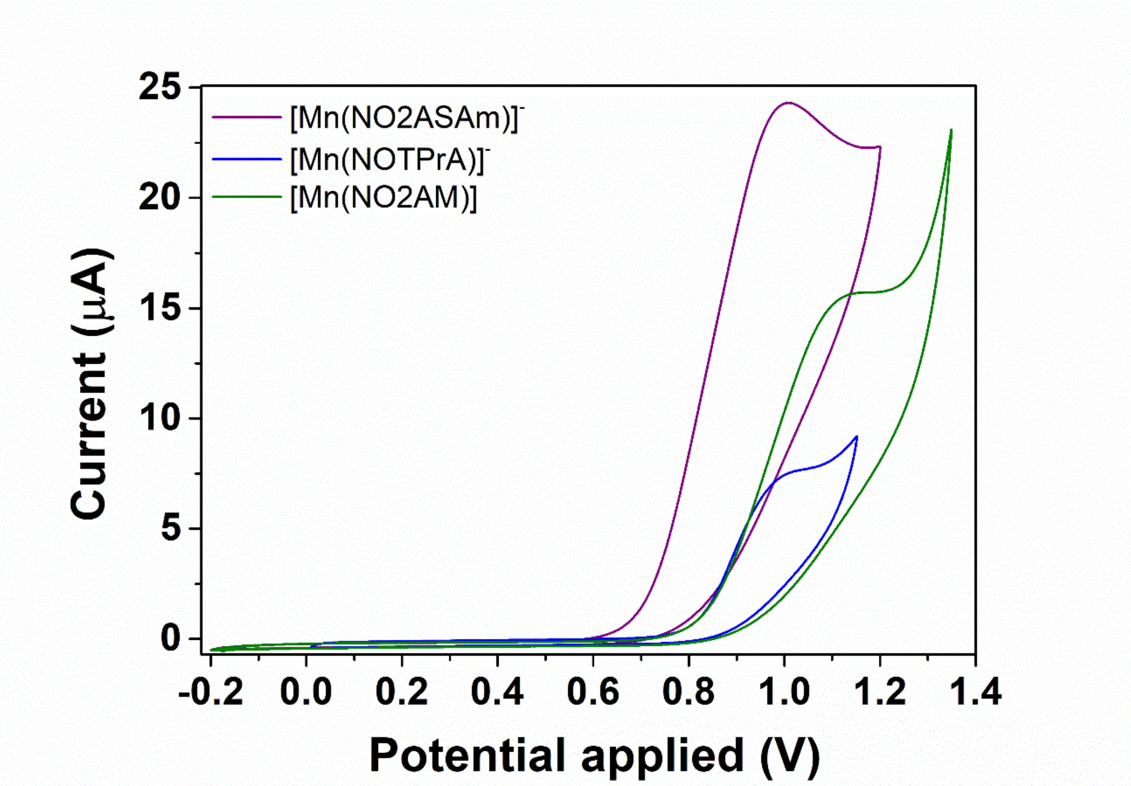

Figure S2. Cyclic voltammogram of **[Mn(NO2ASAm)]⁻**, **[Mn(NOTPrA)]⁻** and **[Mn(NO2AM)]** complexes in aqueous solution in 0.15 M NaCl, recorded at 10 mV s^{-1}.

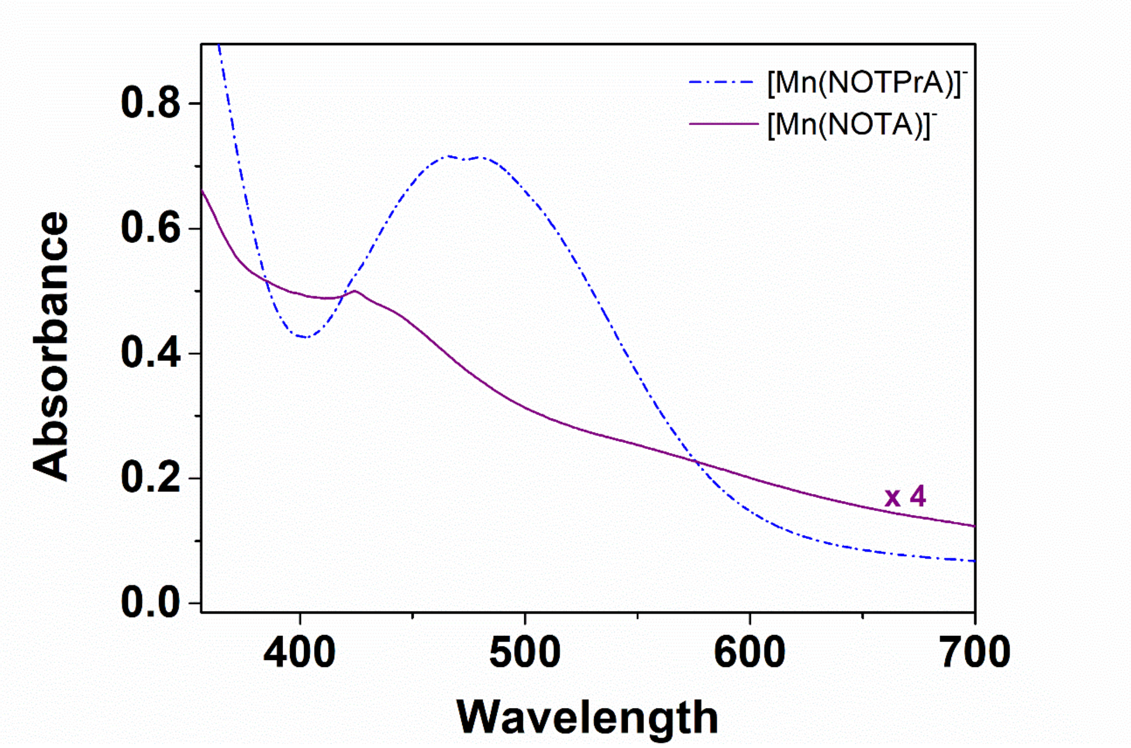

Figure S3. Solid line: Absorption spectrum of a 0.1015 M solution of the Mn^{2+} complex of $NOTA^{3-}$ recorded at 298 K. Dashed line: Absorption spectrum of a 0.1006 M solution of the Mn^{2+} complex of $NOTPrA^{3-}$ recorded at 298 K.

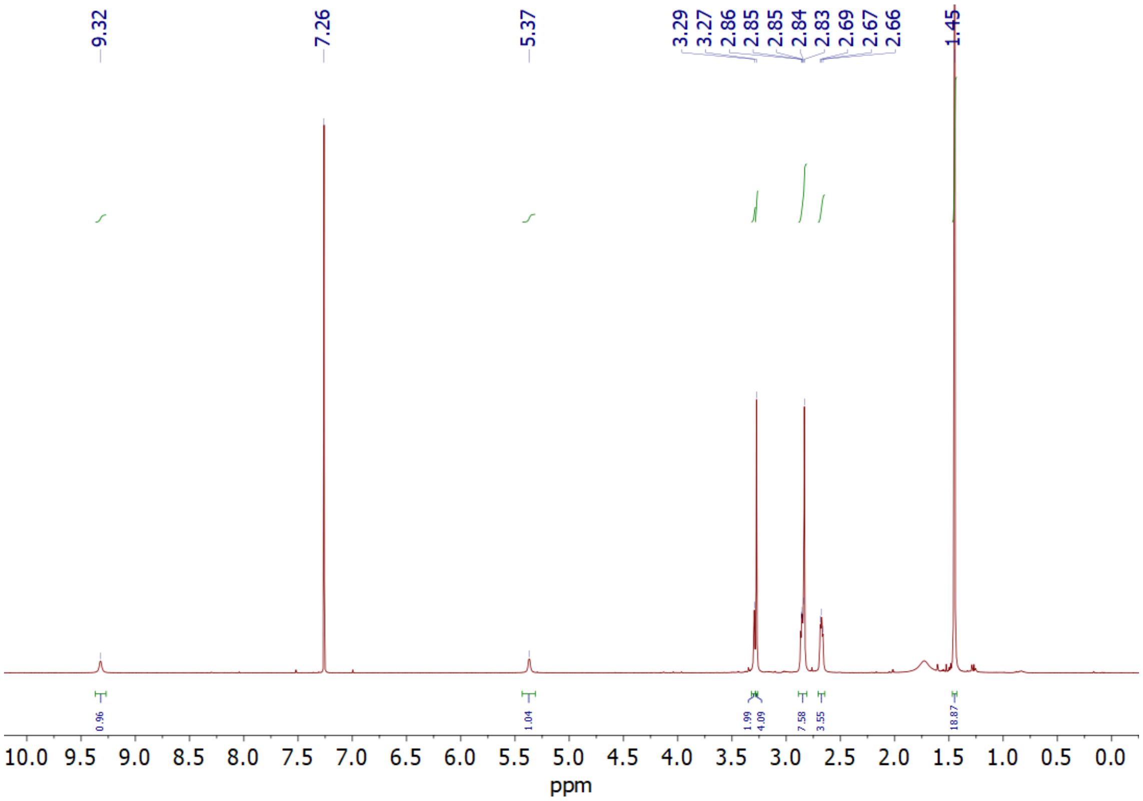

Figure S4. ^{1}H NMR spectrum of compound **1** (400 MHz, $CDCl_3$, 298 K).

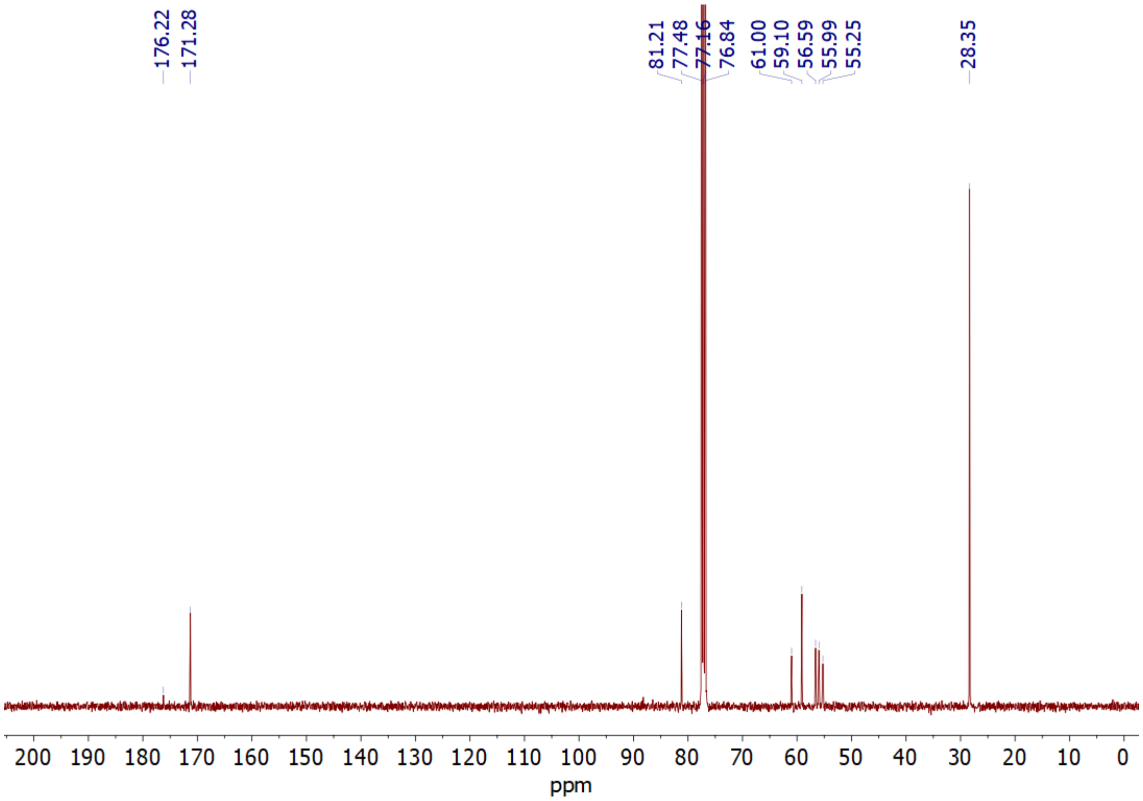

Figure S5. ^{13}C NMR spectrum of compound **1** (101 MHz, $CDCl_3$, 298 K).

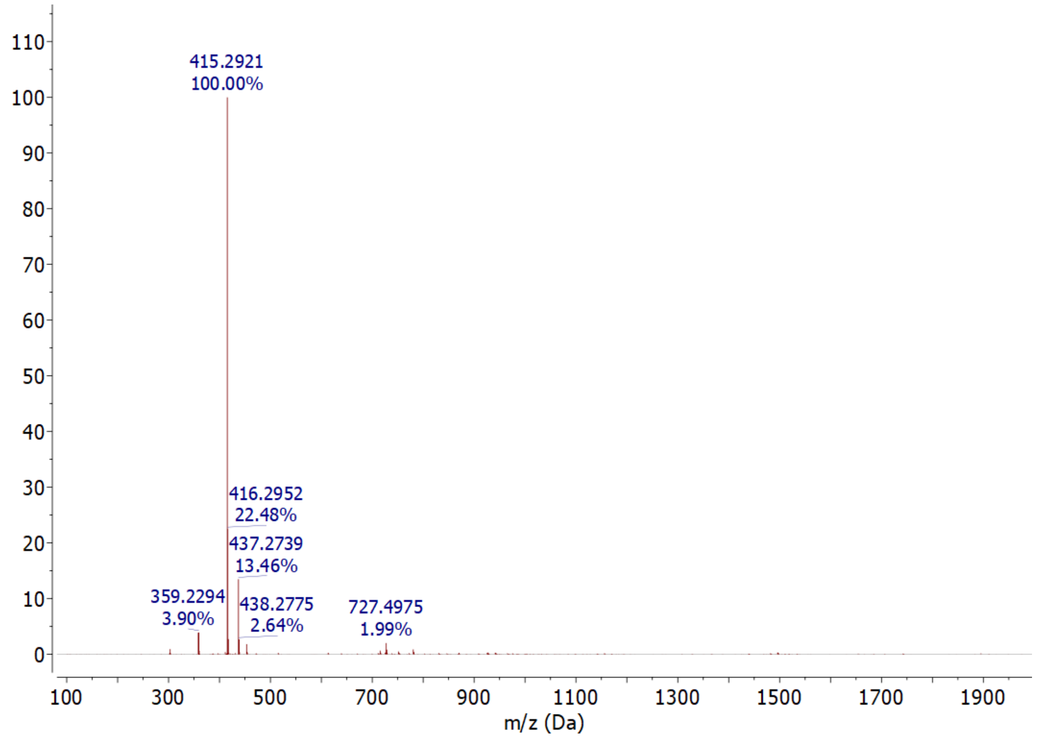

Figure S6. Experimental high resolution mass spectrum (ESI^+) of compound **1**.

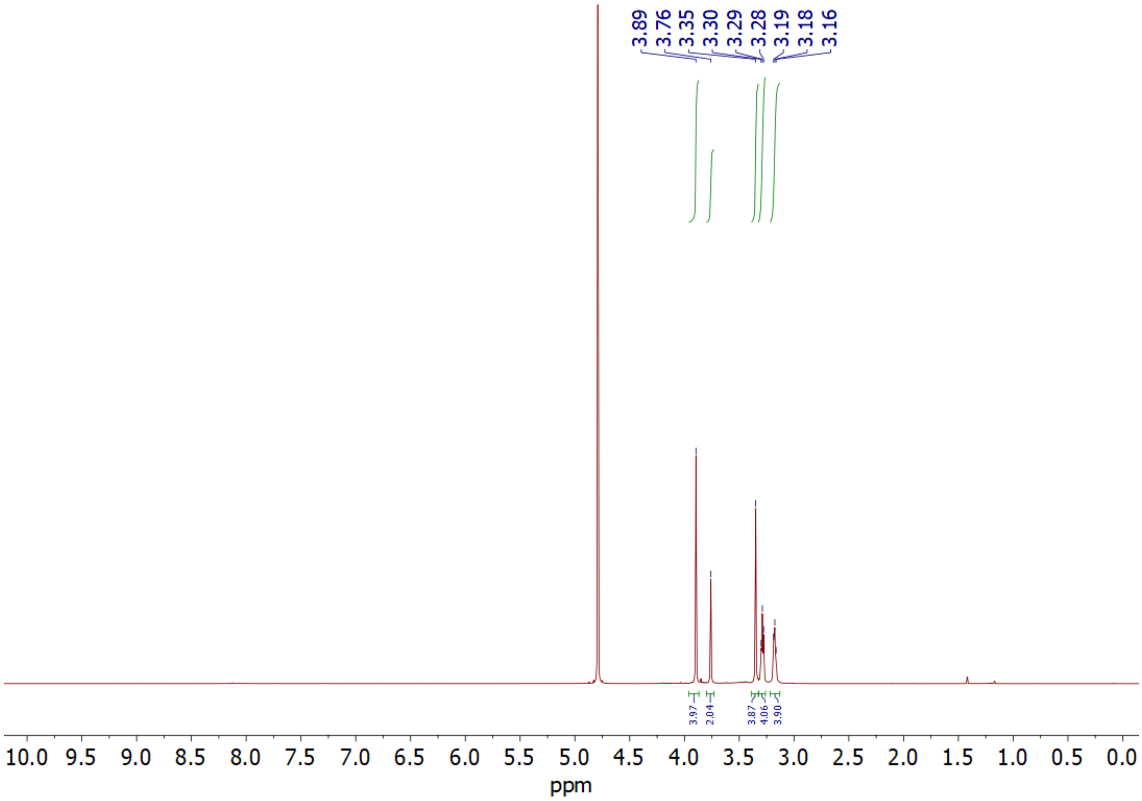

Figure S7. ^{1}H NMR spectrum of **H2NO2AM** (500 MHz, D_2O, pH 1.64, 298 K).

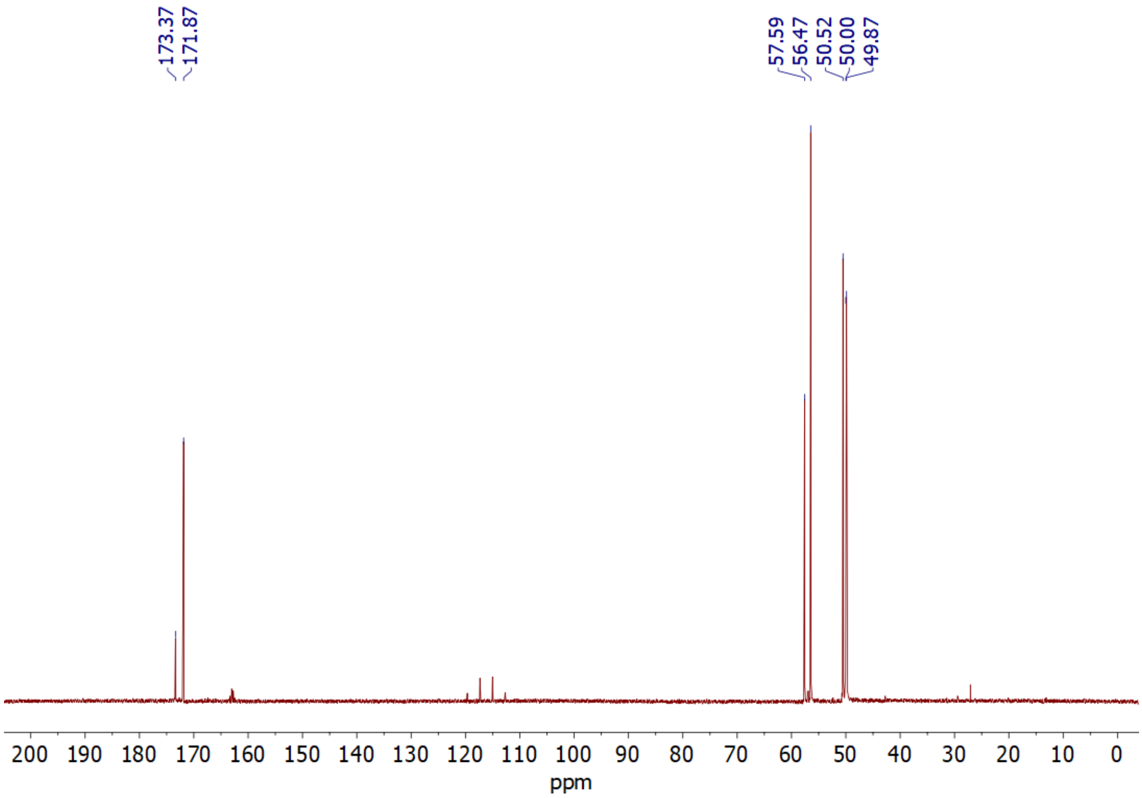

Figure S8. ^{13}C NMR spectrum of **H2NO2AM** (126 MHz, D_2O, pH 1.64, 298 K).

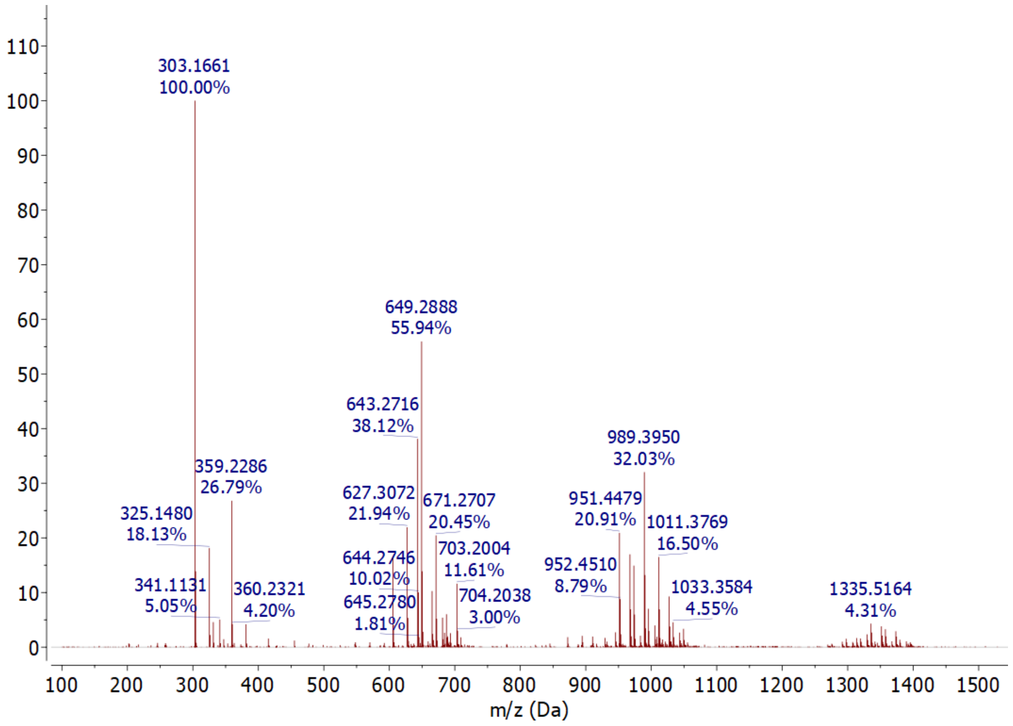

Figure S9. Experimental high resolution mass spectrum (ESI$^+$) of **H$_2$NO2AM**.

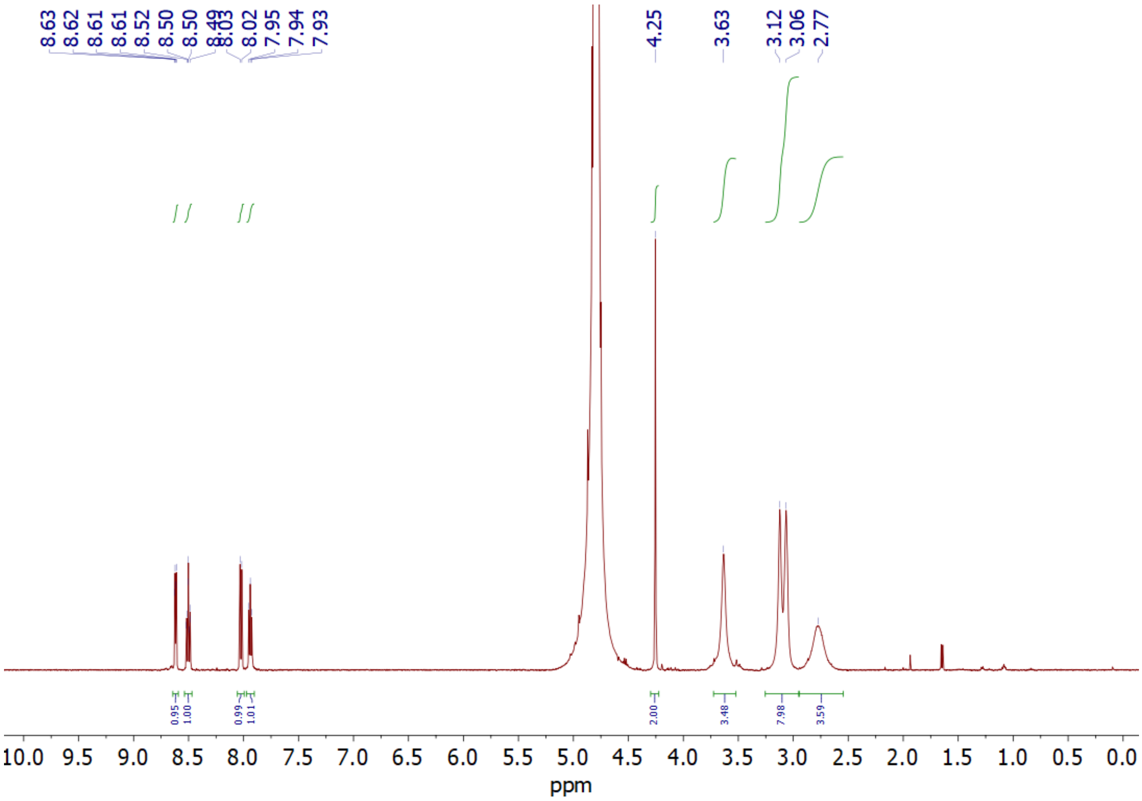

Figure S10. ^{1}H NMR spectrum of **H_2NO2APy** (500 MHz, D_2O, pH 1.23, 298 K).

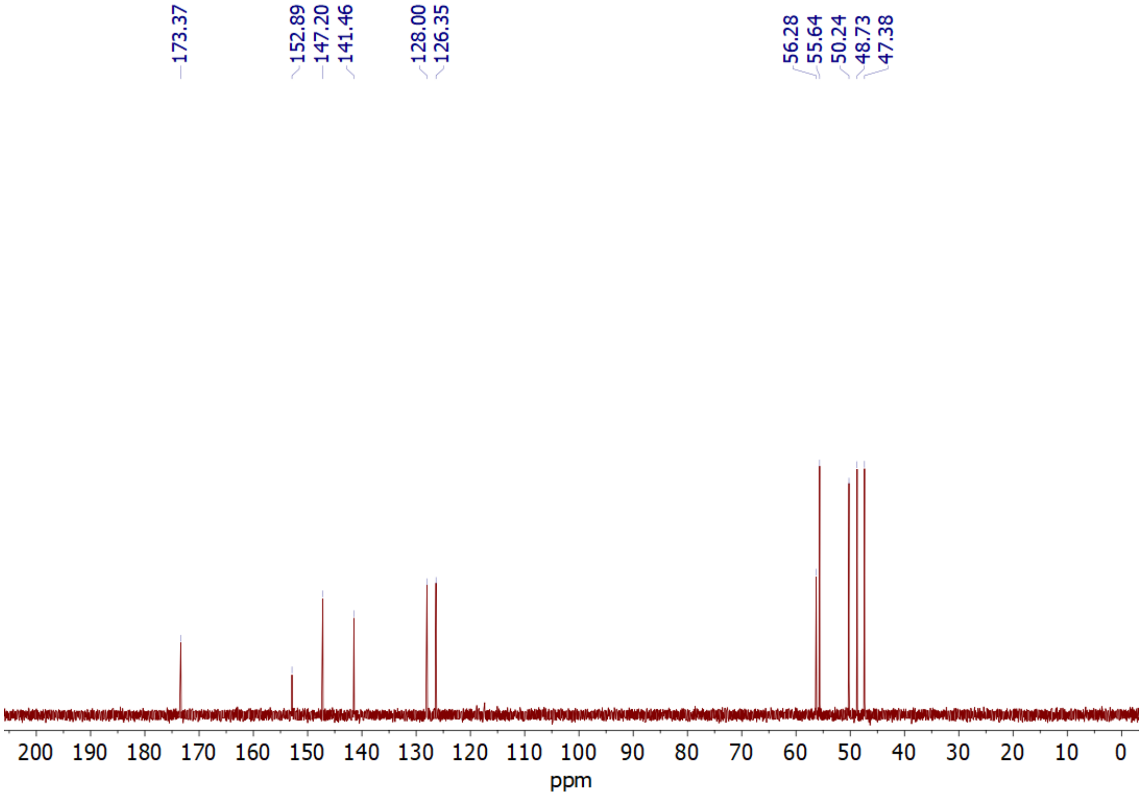

Figure S11. ^{13}C NMR spectrum of **H_2NO2APy** (126 MHz, D_2O, pH 1.23, 298 K).

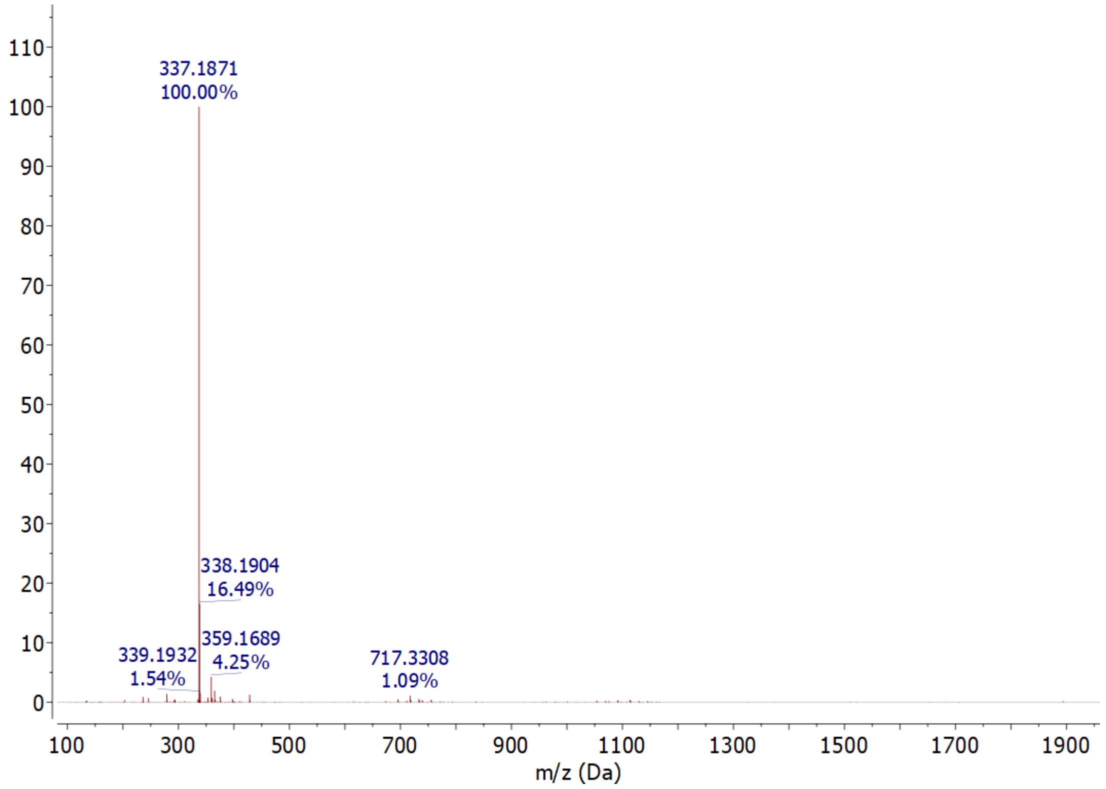

Figure S12. Experimental high resolution mass spectrum (ESI^+) of **H_2NO2APy**.

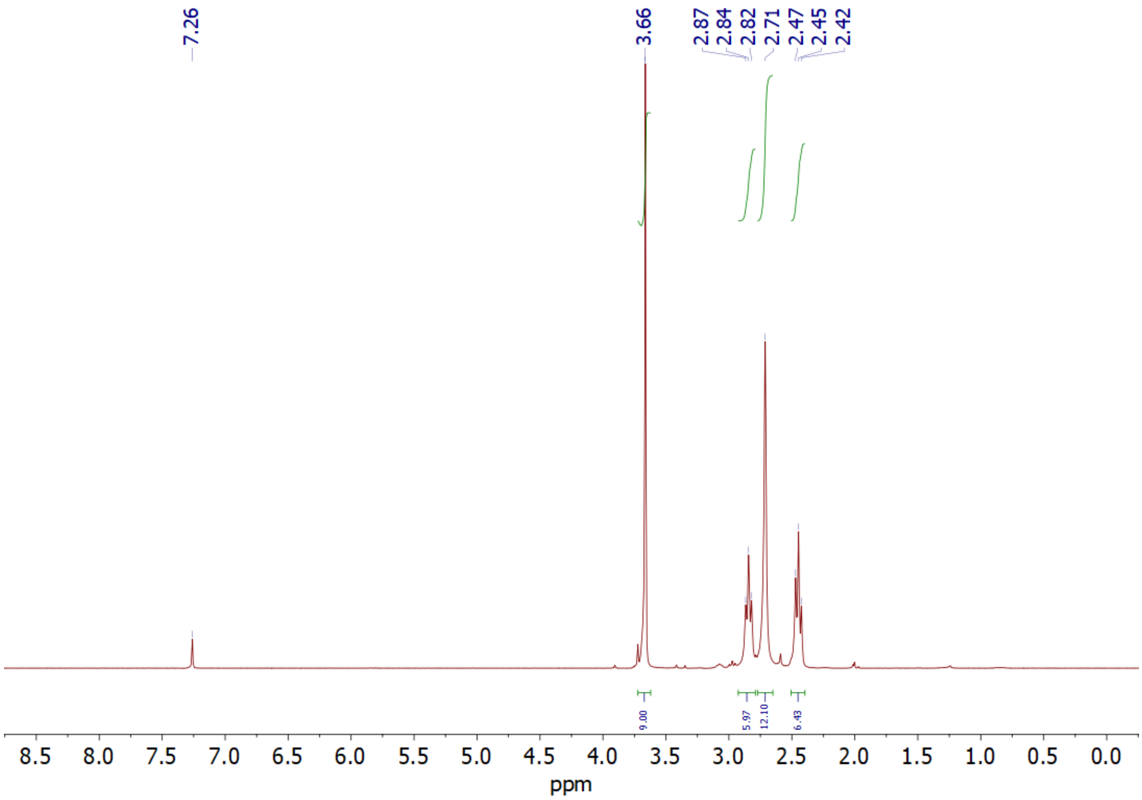

Figure S13. ^{1}H NMR spectrum of compound **2** (300 MHz, $CDCl_3$, 298 K).

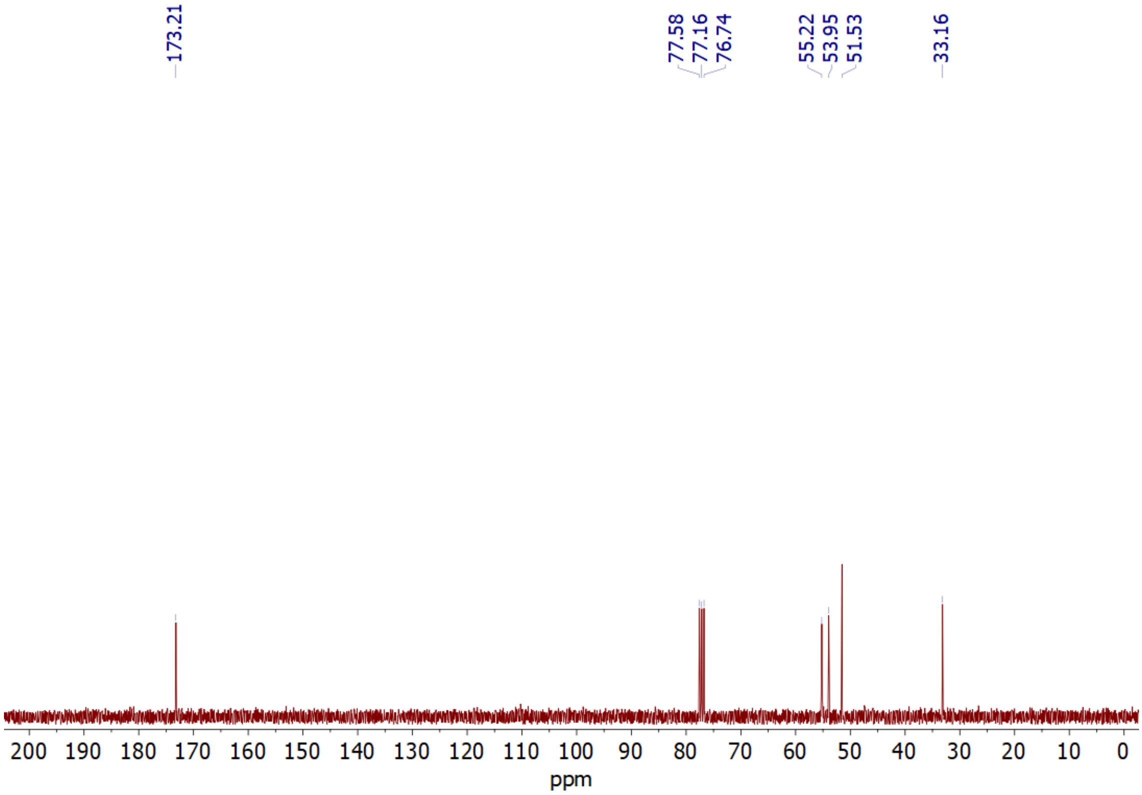

Figure S14. ^{13}C NMR spectrum of compound **2** (75.5 MHz, $CDCl_3$, 298 K).

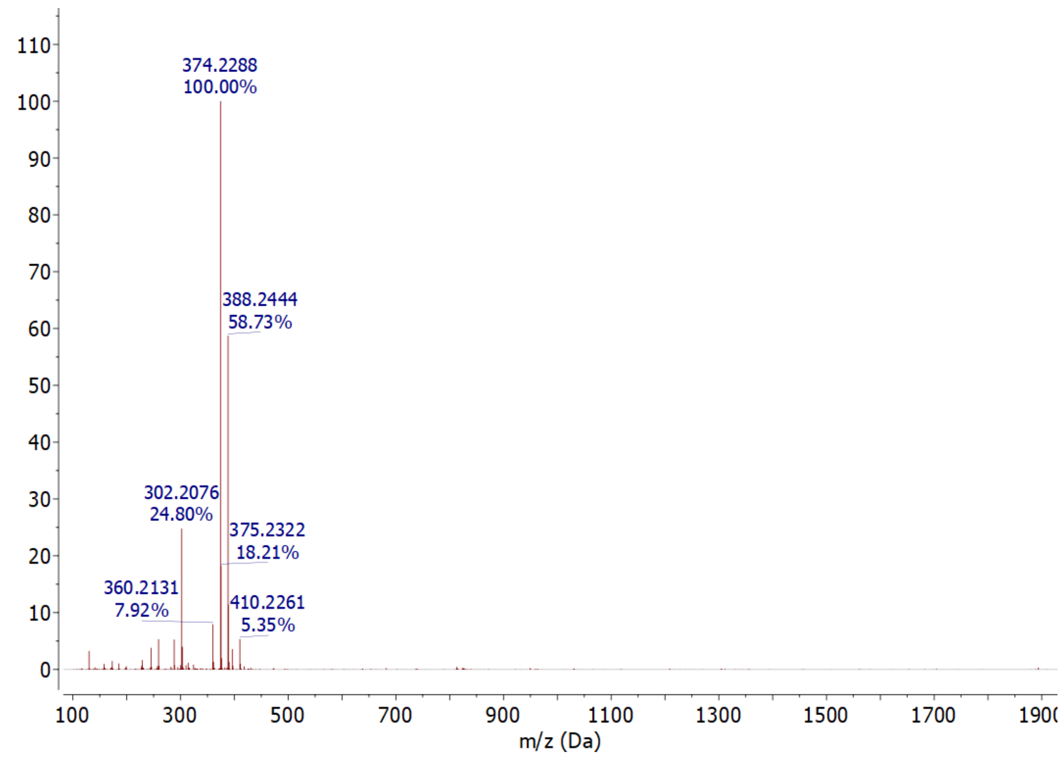

Figure S15. Experimental high resolution mass spectrum (ESI^+) of compound **2.**

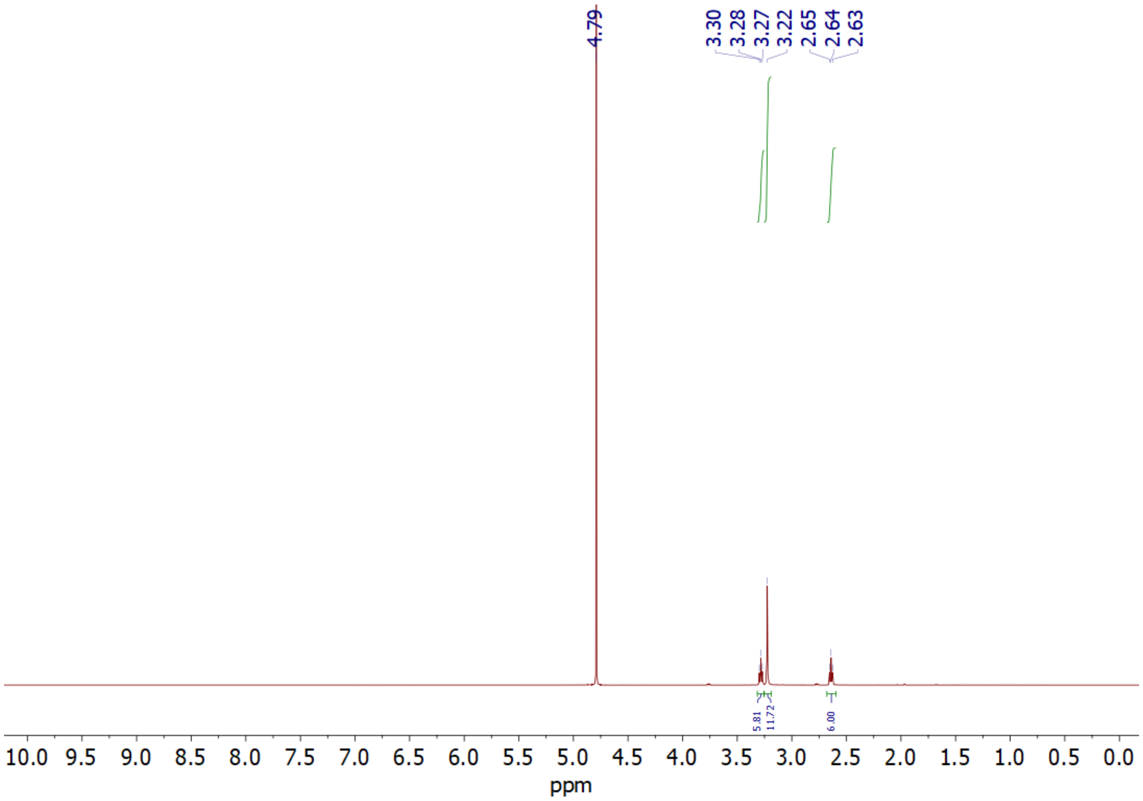

Figure S16. ^{1}H NMR spectrum of **H$_3$NOTPrA** (500 MHz, D_2O, pH 0.58, 298 K).

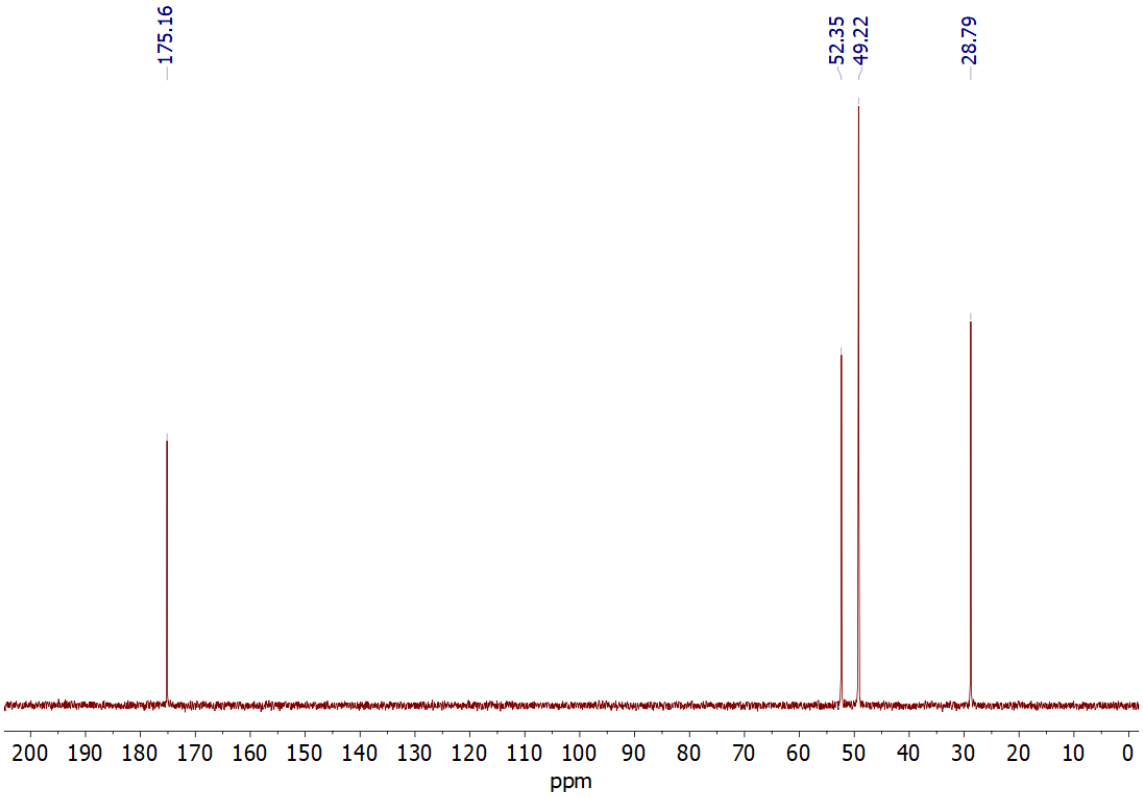

Figure S17. ^{13}C NMR spectrum of **H$_3$NOTPrA** (126 MHz, D_2O, pH 0.58, 298 K).

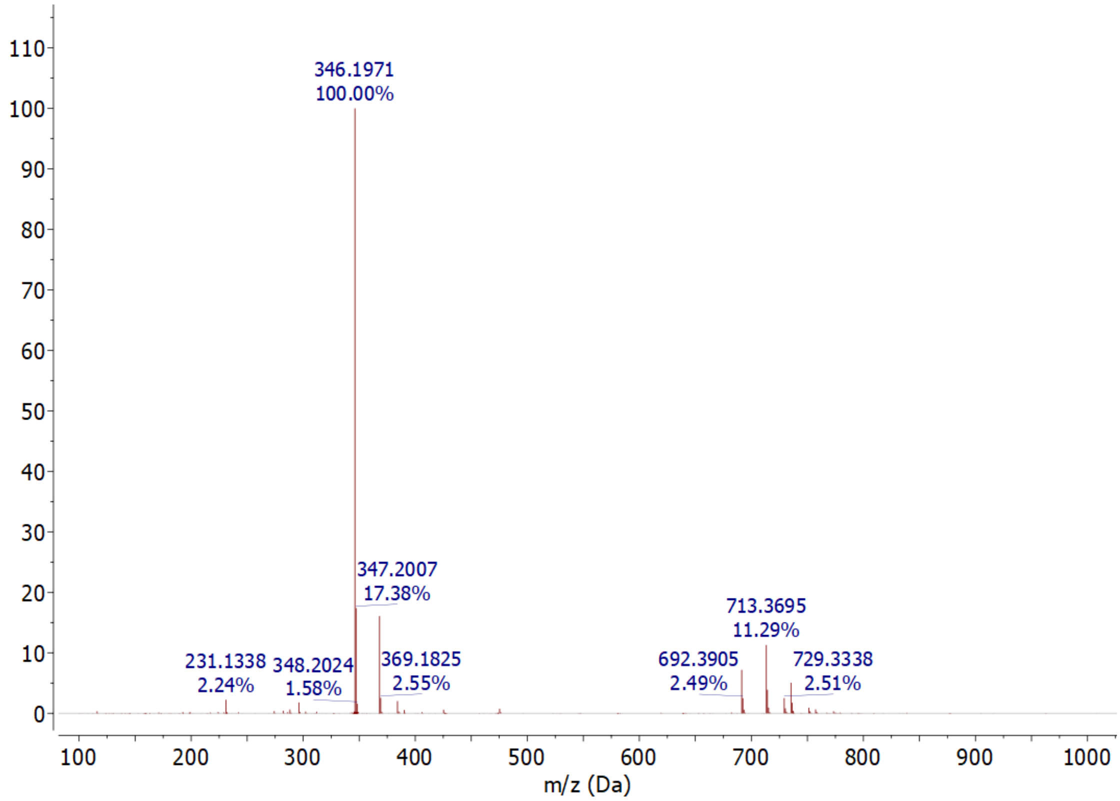

Figure S18. Experimental high resolution mass spectrum (ESI$^+$) of **H$_3$NOTPrA**.

Table S1. Hydration numbers estimated with the relaxivities observed at 0.01 MHz and 298 K.

	r_{1p}	MW	q
$[Mn(NOTA)]^-$	1.76	355.23	0.29
[Mn(NO2AM)]	2.27	355.25	0.38
[Mn(NO2APy)]	1.85	389.31	0.29
$[Mn(NO2ASAm)]^-$	1.95	549.42	0.26
$[Mn(NOTPrA)]^-$	2.77	397.31	0.44

Table S2. Individual contributions to the *D* values obtained with NEVPT2 calculations for **$[Mn(NOTA)]^-$**.

Multiplicity	root	Wave function	D / cm^{-1}	E/D / cm^{-1}
6	0	$1.0\left|d_{xy}^1 d_{yz}^1 d_{z^2}^1 d_{xz}^1 d_{x^2-y^2}^1\right|$	0	0
4	0	$0.53\left|d_{xy}^1 d_{yz}^1 d_{z^2}^2 d_{xz}^0 d_{x^2-y^2}^1\right|$	0.571	0.005
4	1	$0.52\left|d_{xy}^1 d_{yz}^0 d_{z^2}^2 d_{xz}^1 d_{x^2-y^2}^1\right|$	-0.277	0.286
4	2	$0.23\left|d_{xy}^2 d_{yz}^0 d_{z^2}^1 d_{xz}^1 d_{x^2-y^2}^1\right|$<br>$0.23\left|d_{xy}^1 d_{yz}^1 d_{z^2}^1 d_{xz}^0 d_{x^2-y^2}^2\right|$	-0.210	-0.209
4	9	$0.33\left|d_{xy}^2 d_{yz}^1 d_{z^2}^1 d_{xz}^1 d_{x^2-y^2}^0\right|$<br>$0.30\left|d_{xy}^0 d_{yz}^1 d_{z^2}^1 d_{xz}^1 d_{x^2-y^2}^2\right|$	-0.563	-0.563
4	13	$0.27\left|d_{xy}^1 d_{yz}^1 d_{z^2}^1 d_{xz}^1 d_{x^2-y^2}^1\right|$<br>$0.24\left|d_{xy}^1 d_{yz}^2 d_{z^2}^0 d_{xz}^1 d_{x^2-y^2}^1\right|$	-0.180	0.184
4	14	$0.23\left|d_{xy}^1 d_{yz}^1 d_{z^2}^0 d_{xz}^2 d_{x^2-y^2}^1\right|$<br>$0.13\left|d_{xy}^1 d_{yz}^0 d_{z^2}^1 d_{xz}^2 d_{x^2-y^2}^1\right|$<br>$0.13\left|d_{xy}^1 d_{yz}^2 d_{z^2}^1 d_{xz}^0 d_{x^2-y^2}^1\right|$	0.319	0.003
4	15	$0.21\left|d_{xy}^0 d_{yz}^2 d_{z^2}^1 d_{xz}^1 d_{x^2-y^2}^1\right|$<br>$0.21\left|d_{xy}^1 d_{yz}^1 d_{z^2}^1 d_{xz}^2 d_{x^2-y^2}^0\right|$	0.690	0.006
4	16	$0.21\left|d_{xy}^1 d_{yz}^2 d_{z^2}^1 d_{xz}^1 d_{x^2-y^2}^0\right|$<br>$0.21\left|d_{xy}^0 d_{yz}^1 d_{z^2}^1 d_{xz}^2 d_{x^2-y^2}^1\right|$	-0.311	0.322

Table S3. Major individual contributions to the *D* values obtained with NEVPT2 calculations for **[Mn(NOTPrA)]**$^{-}$.

Multiplicity	root	Wave function	*D* / cm^{-1}	*E/D* / cm^{-1}
6	0	$1.0\left\|d_{xy}^1 d_{yz}^1 d_{z^2}^1 d_{xz}^1 d_{x^2-y^2}^1\right\|$	0	0
4	0	$0.19\left\|d_{xy}^2 d_{yz}^0 d_{z^2}^1 d_{xz}^1 d_{x^2-y^2}^1\right\|$<br>$0.13\left\|d_{xy}^1 d_{yz}^1 d_{z^2}^1 d_{xz}^2 d_{x^2-y^2}^0\right\|$<br>$0.13\left\|d_{xy}^2 d_{yz}^1 d_{z^2}^1 d_{xz}^1 d_{x^2-y^2}^0\right\|$<br>$0.12\left\|d_{xy}^1 d_{yz}^1 d_{z^2}^1 d_{xz}^0 d_{x^2-y^2}^2\right\|$	-0.366	-0.308
4	1	$0.37\left\|d_{xy}^1 d_{yz}^0 d_{z^2}^2 d_{xz}^1 d_{x^2-y^2}^1\right\|$	0.631	0.023
4	2	$0.36\left\|d_{xy}^1 d_{yz}^1 d_{z^2}^2 d_{xz}^0 d_{x^2-y^2}^1\right\|$	-0.271	0.278
4	14	$0.46\left\|d_{xy}^1 d_{yz}^1 d_{z^2}^0 d_{xz}^2 d_{x^2-y^2}^1\right\|$	-0.073	0.326
4	15	$0.46\left\|d_{xy}^1 d_{yz}^2 d_{z^2}^0 d_{xz}^1 d_{x^2-y^2}^1\right\|$	0.569	0.129
4	16	$0.19\left\|d_{xy}^0 d_{yz}^2 d_{z^2}^1 d_{xz}^1 d_{x^2-y^2}^1\right\|$<br>$0.16\left\|d_{xy}^1 d_{yz}^1 d_{z^2}^1 d_{xz}^2 d_{x^2-y^2}^0\right\|$<br>$0.16\left\|d_{xy}^1 d_{yz}^2 d_{z^2}^1 d_{xz}^0 d_{x^2-y^2}^1\right\|$<br>$0.15\left\|d_{xy}^1 d_{yz}^0 d_{z^2}^1 d_{xz}^2 d_{x^2-y^2}^1\right\|$	-0.488	-0.449

Table S4. Optimized Cartesian coordinates obtained for **[Mn(NOTA)]⁻** (TPSSh/Def2-TZVPP, scrf=pcm).

Center Number	Atomic Number	Coordinates (Angstroms) X	Y	Z
1	25	0.001271	0.001257	-0.683692
2	8	-1.290390	-1.350283	-1.665153
3	8	-2.264305	-3.370056	-1.670789
4	8	-0.535693	1.792507	-1.664783
5	8	-1.805990	3.641222	-1.663373
6	7	-0.694047	-1.517070	0.999343
7	7	-0.962140	1.358285	1.003558
8	7	1.662951	0.152129	0.998297
9	6	1.720730	-1.225718	1.524957
10	1	2.454999	-1.314046	2.338021
11	1	2.060561	-1.862969	0.704799
12	6	0.360354	-1.716480	2.019110
13	1	0.443646	-2.772444	2.291821
14	1	0.076865	-1.191132	2.931058
15	6	-1.914029	-0.878898	1.532228
16	1	-2.354015	-1.472557	2.345834
17	1	-2.638581	-0.853216	0.714511
18	6	-1.656394	0.543894	2.026411
19	1	-2.610733	0.999973	2.304888
20	1	-1.054440	0.525900	2.934837
21	6	0.201899	2.095702	1.532797
22	1	-0.088700	2.772561	2.348509
23	1	0.582798	2.711205	0.714011
24	6	1.308068	1.161131	2.021622
25	1	2.181092	1.760691	2.295322
26	1	0.996745	0.649602	2.932311
27	6	2.893508	0.487951	0.269237
28	1	3.793168	0.200711	0.826205
29	1	2.924858	1.572213	0.132229
30	6	2.943425	-0.132040	-1.138342
31	6	-1.023930	-2.748444	0.268286
32	1	-1.719480	-3.385084	0.827373
33	1	-0.102348	-3.318679	0.124153
34	6	-1.593420	-2.476227	-1.135118
35	6	-1.868621	2.258543	0.277380
36	1	-2.068408	3.179411	0.837557
37	1	-2.823942	1.744797	0.140532
38	6	-1.359463	2.615251	-1.130270
39	8	1.817751	-0.434466	-1.669210
40	8	4.052881	-0.258893	-1.675424

E(UTPSSh) = -2235.5529445 Hartree

Zero-point correction = 0.314885
Thermal correction to Energy = 0.335574
Thermal correction to Enthalpy= 0.336518
Thermal correction to Gibbs Free Energy = 0.264901
Sum of electronic and zero-point Energies = -2235.238060
Sum of electronic and thermal Energies = -2235.217371
Sum of electronic and thermal Enthalpies = -2235.216427
Sum of electronic and thermal Free Energies = -2235.288044

Table S5. Optimized Cartesian coordinates obtained for **[Mn(NOTPrA)]**⁻ (TPSSh/Def2-TZVPP, scrf=pcm).

Center Number	Atomic Number	Coordinates (Angstroms) X	Y	Z
1	25	0.000544	0.000692	-0.528058
2	8	1.661790	-0.766274	-1.549356
3	8	3.505255	-1.861411	-2.165548
4	7	0.502093	-1.602359	1.130580
5	6	1.407127	-1.034329	2.156471
6	6	2.087295	0.253064	1.690155
7	6	2.540867	-1.686599	-1.400855
8	6	2.430177	-2.619688	-0.187140
9	6	1.063541	-2.801602	0.465759
10	1	2.179728	-1.757679	2.435084
11	1	0.834742	-0.848182	3.065406
12	1	2.797498	0.026994	0.893621
13	1	2.655860	0.680590	2.528795
14	1	3.168354	-2.281581	0.547570
15	1	2.776103	-3.605224	-0.505881
16	1	0.335844	-3.108753	-0.289014
17	1	1.141150	-3.619667	1.197854
18	8	-1.497068	-1.042665	-1.555419
19	8	-3.363659	-2.097697	-2.172146
20	7	-1.639540	0.362575	1.132602
21	6	-1.599351	-0.708114	2.155295
22	6	-0.825497	-1.939565	1.684410
23	6	-2.731951	-1.350058	-1.405774
24	6	-3.484928	-0.793641	-0.189383
25	6	-2.959203	0.478083	0.468890
26	1	-2.611705	-1.015939	2.434844
27	1	-1.150055	-0.308161	3.064468
28	1	-1.376933	-2.438746	0.886444
29	1	-0.739372	-2.648687	2.520532
30	1	-3.561485	-1.605284	0.541596
31	1	-4.511457	-0.598857	-0.507119
32	1	-2.861664	1.264984	-0.282623
33	1	-3.706342	0.816576	1.202690
34	8	-0.155622	1.819708	-1.554467
35	8	-0.131282	3.964613	-2.167821
36	7	1.132529	1.234933	1.135717
37	6	0.185891	1.732805	2.160357
38	6	-1.267788	1.679295	1.690291
39	6	0.196658	3.042146	-1.401694
40	6	1.051377	3.413774	-0.181937
41	6	1.891255	2.322316	0.474367
42	1	0.425784	2.762855	2.441901
43	1	0.308139	1.142011	3.068395
44	1	-1.425029	2.408798	0.894601
45	1	-1.924357	1.956603	2.527801
46	1	0.384065	3.882049	0.549145
47	1	1.732072	4.208115	-0.495504
48	1	2.524736	1.846945	-0.278093
49	1	2.557030	2.799392	1.209439

E(UTPSSh) = -2353.5324751 Hartree

Zero-point correction = 0.400399

```
Thermal correction to Energy = 0.424836
Thermal correction to Enthalpy = 0.425780
Thermal correction to Gibbs Free Energy = 0.345097
Sum of electronic and zero-point Energies = -2353.132076
Sum of electronic and thermal Energies = -2353.107639
Sum of electronic and thermal Enthalpies = -2353.106695
Sum of electronic and thermal Free Energies = -2353.187378
```

Table S6. Optimized Cartesian coordinates obtained for **[Mn(NO2AM)]** (TPSSh/Def2-TZVPP, scrf=pcm).

Center Number	Atomic Number	Coordinates (Angstroms) X	Y	Z
1	25	0.043827	0.006561	-0.665941
2	8	0.927293	1.581403	-1.719185
3	8	1.488997	3.751379	-1.749660
4	8	0.756283	-1.682479	-1.680441
5	8	2.341694	-3.264290	-1.790945
6	7	0.489437	1.614157	0.988623
7	7	1.289174	-1.154249	0.939799
8	7	-1.521905	-0.454689	1.082166
9	6	-1.803182	0.888939	1.636880
10	1	-2.492579	0.830326	2.489158
11	1	-2.300108	1.462230	0.850208
12	6	-0.526799	1.611093	2.065572
13	1	-0.784275	2.631827	2.361009
14	1	-0.104618	1.138524	2.951971
15	6	1.836381	1.221020	1.454049
16	1	2.191030	1.881914	2.256246
17	1	2.510441	1.344779	0.603035
18	6	1.882784	-0.229986	1.934356
19	1	2.921565	-0.499978	2.142257
20	1	1.348694	-0.334968	2.878534
21	6	0.309419	-2.094886	1.519238
22	1	0.760433	-2.702950	2.314976
23	1	0.011729	-2.773309	0.716371
24	6	-0.924345	-1.385613	2.073396
25	1	-1.653399	-2.136969	2.387436
26	1	-0.668784	-0.821989	2.969539
27	6	-2.709615	-1.017988	0.439375
28	1	-3.612280	-0.910954	1.052825
29	1	-2.548766	-2.086656	0.276500
30	6	-2.911370	-0.403247	-0.937659
31	6	0.537009	2.890997	0.257888
32	1	1.141019	3.639316	0.782790
33	1	-0.480989	3.283153	0.188399
34	6	1.045066	2.743443	-1.186480
35	6	2.297893	-1.878550	0.150659
36	1	2.700657	-2.741565	0.692468
37	1	3.129637	-1.198577	-0.050761
38	6	1.759647	-2.335852	-1.216993
39	7	-4.145645	-0.418023	-1.431869
40	8	-1.946447	0.054723	-1.576981
41	1	-4.318677	-0.085719	-2.368799
42	1	-4.923720	-0.774954	-0.900659

E(UTPSSh) = -2216.1351774 Hartree
Zero-point correction = 0.340117
Thermal correction to Energy = 0.361475
Thermal correction to Enthalpy = 0.362419
Thermal correction to Gibbs Free Energy = 0.289434
Sum of electronic and zero-point Energies = -2215.795060
Sum of electronic and thermal Energies = -2215.773702
Sum of electronic and thermal Enthalpies = -2215.772758
Sum of electronic and thermal Free Energies = -2215.845743

Table S7. Optimized Cartesian coordinates obtained for **[Mn(NO2APy)]** (TPSSh/Def2-TZVPP, scrf=pcm).

Center Number	Atomic Number	Coordinates (Angstroms) X	Y	Z
1	25	-0.158898	0.024861	0.495235
2	8	-0.248003	1.809740	1.579045
3	8	-0.419409	4.044589	1.542277
4	8	-0.649895	-1.384061	1.969883
5	8	-2.263000	-2.603496	2.939506
6	7	-1.025028	1.589311	-1.037881
7	7	-2.143407	-0.954529	-0.267955
8	7	0.408961	-0.838041	-1.634439
9	6	0.629850	0.375405	-2.450997
10	1	0.865742	0.117584	-3.492010
11	1	1.500064	0.887576	-2.034178
12	6	-0.578098	1.312046	-2.422319
13	1	-0.316466	2.239224	-2.939586
14	1	-1.406122	0.878495	-2.982411
15	6	-2.483219	1.441246	-0.851509
16	1	-3.042030	2.107415	-1.522762
17	1	-2.702275	1.749624	0.173759
18	6	-2.950744	0.001379	-1.061741
19	1	-4.008717	-0.069694	-0.795345
20	1	-2.886208	-0.267899	-2.115842
21	6	-1.658564	-2.109096	-1.050856
22	1	-2.491860	-2.668928	-1.496987
23	1	-1.152972	-2.775885	-0.348414
24	6	-0.686284	-1.695475	-2.153334
25	1	-0.281456	-2.595209	-2.624713
26	1	-1.211338	-1.154923	-2.939649
27	6	1.654217	-1.601601	-1.460031
28	1	2.153489	-1.787067	-2.419016
29	1	1.387506	-2.576461	-1.041857
30	6	-0.562369	2.894488	-0.541067
31	1	-1.214615	3.711634	-0.868603
32	1	0.432384	3.084977	-0.952498
33	6	-0.414040	2.937883	0.989362
34	6	-2.824916	-1.406232	0.954825
35	1	-3.540639	-2.210859	0.752408
36	1	-3.385440	-0.563208	1.367067
37	6	-1.840448	-1.856918	2.047837
38	6	2.614038	-0.932619	-0.504492
39	6	3.989894	-1.104273	-0.619401
40	6	2.883183	0.361330	1.394720
41	6	4.824535	-0.523874	0.326827
42	1	4.394105	-1.684340	-1.439380
43	6	4.261769	0.223881	1.356157
44	1	2.393462	0.944611	2.164826
45	1	5.898127	-0.647347	0.258054
46	1	4.875237	0.697688	2.110589
47	7	2.074062	-0.207900	0.488291

E(UTPSSh) =-2294.5539235
Zero-point correction= 0.382499 (Hartree/Particle)
Thermal correction to Energy= 0.405027

```
Thermal correction to Enthalpy=                 0.405972
Thermal correction to Gibbs Free Energy=        0.330409
Sum of electronic and zero-point Energies=         -2294.171424
Sum of electronic and thermal Energies=            -2294.148896
Sum of electronic and thermal Enthalpies=          -2294.147952
Sum of electronic and thermal Free Energies=       -2294.223514
```

Table S8. Optimized Cartesian coordinates obtained for **[Mn(NO2ASAm)]⁻** (TPSSh/Def2-TZVPP, scrf=pcm).

Center Number	Atomic Number	Coordinates (Angstroms) X	Y	Z
1	25	-1.709221	0.131669	-0.285559
2	16	0.898553	2.269420	-0.448709
3	9	5.914276	-2.174382	-0.065401
4	9	6.988835	-0.453424	-0.830184
5	9	6.684621	-0.675427	1.305880
6	8	0.526327	2.159818	-1.851714
7	8	1.201950	3.611945	0.046490
8	8	-0.847406	-0.965645	-1.878351
9	8	0.337930	-2.754346	-2.529666
10	8	-2.900756	1.474303	-1.406069
11	8	-4.865000	1.955782	-2.374946
12	7	-1.674708	-2.191206	0.402559
13	7	-3.996573	-0.512984	0.108337
14	7	-1.966405	0.117046	2.082618
15	7	-0.175876	1.518232	0.428170
16	6	-1.156242	-1.029281	2.537613
17	1	-1.200011	-1.146123	3.629688
18	1	-0.119264	-0.818648	2.272087
19	6	-1.583512	-2.336399	1.870842
20	1	-0.869672	-3.120076	2.142277
21	1	-2.549310	-2.662596	2.256196
22	6	-2.923710	-2.736162	-0.160011
23	1	-3.041698	-3.802305	0.081122
24	1	-2.845364	-2.650449	-1.246195
25	6	-4.153326	-1.968805	0.319344
26	1	-5.034330	-2.344972	-0.208694
27	1	-4.332725	-2.160739	1.377104
28	6	-4.328324	0.291393	1.298948
29	1	-5.367391	0.127703	1.619063
30	1	-4.235741	1.340538	1.007026
31	6	-3.391902	0.004228	2.470275
32	1	-3.622830	0.698710	3.284017
33	1	-3.575643	-0.994211	2.866231
34	6	-1.403671	1.407060	2.527983
35	1	-1.302379	1.434114	3.622290
36	1	-2.118065	2.180009	2.236605
37	6	-0.061171	1.711638	1.878686
38	1	0.200852	2.744694	2.125080
39	1	0.723360	1.073467	2.306966
40	6	2.444268	1.367470	-0.288918
41	6	2.645223	0.215009	-1.050145
42	1	1.884453	-0.106161	-1.749538
43	6	3.820987	-0.508289	-0.910758
44	1	3.980662	-1.400356	-1.502853
45	6	4.793681	-0.082974	-0.003852
46	6	4.593702	1.065221	0.757979
47	1	5.350311	1.394229	1.458284
48	6	3.416083	1.791992	0.614197
49	1	3.256437	2.693683	1.190075
50	6	6.083176	-0.842997	0.107207
51	6	-0.507372	-2.736335	-0.298699
52	1	-0.548183	-3.829195	-0.384226

53	1	0.388603	-2.485621	0.276012
54	6	-0.325380	-2.120236	-1.695408
55	6	-4.732312	-0.033492	-1.068019
56	1	-5.796057	0.123300	-0.852267
57	1	-4.666189	-0.796461	-1.848312
58	6	-4.134391	1.251232	-1.662280

E(UTPSSh) = -3258.6486279 Hartree
Zero-point correction = 0.440890
Thermal correction to Energy = 0.472744
Thermal correction to Enthalpy = 0.473689
Thermal correction to Gibbs Free Energy = 0.374494
Sum of electronic and zero-point Energies = -3258.207738
Sum of electronic and thermal Energies = -3258.175883
Sum of electronic and thermal Enthalpies = -3258.174939
Sum of electronic and thermal Free Energies = -3258.274134

Table S9. Sample ORCA input file.

```
#
# NEVPT2_NO2A_Am
#
! RIJCOSX tightscf Normalprint cpcm(water) def2-TZVPP Def2/JK moread
GridX6 NoFinalGridX
%moinp "CASSCF_NO2A_Am.gbw"
%pal
  nprocs 6
end
% maxcore 7000
* xyz 0 6
 Mn                0.04382700    0.00656100   -0.66594100
 O                 0.92729300    1.58140300   -1.71918500
 O                 1.48899700    3.75137900   -1.74966000
 O                 0.75628300   -1.68247900   -1.68044100
 O                 2.34169400   -3.26429000   -1.79094500
 N                 0.48943700    1.61415700    0.98862300
 N                 1.28917400   -1.15424900    0.93979900
 N                -1.52190500   -0.45468900    1.08216600
 C                -1.80318200    0.88893900    1.63688000
 H                -2.49257900    0.83032600    2.48915800
 H                -2.30010800    1.46223000    0.85020800
 C                -0.52679900    1.61109300    2.06557200
 H                -0.78427500    2.63182700    2.36100900
 H                -0.10461800    1.13852400    2.95197100
 C                 1.83638100    1.22102000    1.45404900
 H                 2.19103000    1.88191400    2.25624600
 H                 2.51044100    1.34477900    0.60303500
 C                 1.88278400   -0.22998600    1.93435600
 H                 2.92156500   -0.49997800    2.14225700
 H                 1.34869400   -0.33496800    2.87853400
 C                 0.30941900   -2.09488600    1.51923800
 H                 0.76043300   -2.70295000    2.31497600
 H                 0.01172900   -2.77330900    0.71637100
 C                -0.92434500   -1.38561300    2.07339600
 H                -1.65339900   -2.13696900    2.38743600
 H                -0.66878400   -0.82198900    2.96953900
 C                -2.70961500   -1.01798800    0.43937500
 H                -3.61228000   -0.91095400    1.05282500
 H                -2.54876600   -2.08665600    0.27650000
 C                -2.91137000   -0.40324700   -0.93765900
 C                 0.53700900    2.89099700    0.25788800
 H                 1.14101900    3.63931600    0.78279000
 H                -0.48098900    3.28315300    0.18839900
 C                 1.04506600    2.74344300   -1.18648000
 C                 2.29789300   -1.87855000    0.15065900
 H                 2.70065700   -2.74156500    0.69246800
 H                 3.12963700   -1.19857700   -0.05076100
 C                 1.75964700   -2.33585200   -1.21699300
 N                -4.14564500   -0.41802300   -1.43186900
 O                -1.94644700    0.05472300   -1.57698100
 H                -4.31867700   -0.08571900   -2.36879900
 H                -4.92372000   -0.77495400   -0.90065900
*
%cpcm smd true # turn on SMD
smdsolvent "water" # specify the name of solvent from the list
end
%method
```

```
SpecialGridAtoms 25
SpecialGridIntAcc 7
end
%casscf
nel 5
norb 5
mult 6,4,2
nroots 1,24,75
actorbs dorbs
TrafoStep ri
PTMethod FIC_NEVPT2
rel
ninitstates 28
dosoc true
gtensor true
domagnetization true
dosusceptibility true
end
end
```

Table S10. Bond distances (Å) and angles (°) of the metal coordination environment in **$\{[Mn(NO2ASAm)]\}_2[Mn(H_2O)_6]\cdot 2H_2O$**.

	Distances (Å)		Angles (°)
Mn(1)-O(3)	2.1303(8)	O(3)-Mn(1)-N(1)	73.71(3)
Mn(1)-O(5)	2.1219(8)	O(3)-Mn(1)-N(2)	111.75(3)
Mn(1)-N(1)	2.3560(10)	O(3)-Mn(1)-N(3)	144.24(3)
Mn(1)-N(2)	2.3482(9)	O(3)-Mn(1)-N(4)	105.10(3)
Mn(1)-N(3)	2.3331(10)	O(5)-Mn(1)-O(3)	96.20(3)
Mn(1)-N(4)	2.1752(9)	O(5)-Mn(1)-N(1)	141.23(3)
Mn(2)-O(8)	2.1613(9)	O(5)-Mn(1)-N(2)	74.69(3)
Mn(2)-O(8)	2.1613(9)	O(5)-Mn(1)-N(3)	119.15(3)
Mn(2)-O(7)	2.1864(8)	O(5)-Mn(1)-N(4)	101.00(3)
Mn(2)-O(7)	2.1865(8)	N(2)-Mn(1)-N(1)	74.83(3)
Mn(2)-O(9)	2.1756(8)	N(3)-Mn(1)-N(1)	75.01(3)
Mn(2)-O(9)	2.1756(8)	N(3)-Mn(1)-N(2)	75.70(3)
		N(4)-Mn(1)-N(1)	117.76(4)
		N(4)-Mn(1)-N(2)	143.15(4)
		N(4)-Mn(1)-N(3)	75.01(3)
		O(8)-Mn(2)-O(8)	180.0
		O(8)-Mn(2)-O(7)	87.96(4)
		O(8)-Mn(2)-O(7)	92.03(4)
		O(8)-Mn(2)-O(7)	87.97(4)
		O(8)-Mn(2)-O(7)	92.04(4)
		O(8)-Mn(2)-O(9)	92.95(3)
		O(8)-Mn(2)-O(9)	87.05(3)
		O(8)-Mn(2)-O(9)	92.95(3)
		O(8)-Mn(2)-O(9)	87.05(3)
		O(7)-Mn(2)-O(7)	180.0
		O(9)-Mn(2)-O(7)	89.20(3)
		O(9)-Mn(2)-O(7)	90.80(3)
		O(9)-Mn(2)-O(7)	90.80(3)
		O(9)-Mn(2)-O(7)	89.20(3)
		O(9)-Mn(2)-O(9)	180.0
